# ILF3 is a substrate of SPOP for regulating serine biosynthesis in colorectal cancer

**DOI:** 10.1038/s41422-019-0257-1

**Published:** 2019-11-26

**Authors:** Kai Li, Jian-lin Wu, Baifu Qin, Zongmin Fan, Qin Tang, Weisi Lu, Haipeng Zhang, Fan Xing, Manqi Meng, Shaomin Zou, Wenxia Wei, Honglei Chen, Jian Cai, Huaiming Wang, Hui Zhang, Jiayue Cai, Ling Fang, Xiqing Bian, Chuangqi Chen, Ping Lan, Bart Ghesquière, Lekun Fang, Mong-Hong Lee

**Affiliations:** 1 Guangdong Provincial Key laboratory of Colorectal and Pelvic Floor Disease, The Sixth Affiliated Hospital of Sun Yat-sen University, 510655 Guangzhou, China; 2 Guangdong Research Institute of Gastroenterology, The Sixth Affiliated Hospital of Sun Yat-sen University, 510655 Guangzhou, China; 3 State Key Laboratory of Quality Research in Chinese Medicine, Macau Institute for Applied Research in Medicine and Health, Macau University of Science and Technology, Macao, China; 4 State Key Laboratory of Ophthalmology, Zhongshan Ophthalmic Center, Sun Yat-sen University, Guangzhou, China; 5 Department of Pharmacology, School of Medicine, Jinan University, 510632 Guangzhou, China; 6 School of Medicine, Sun Yat-sen University, Guangzhou, China; 7 Department of Colorectal Surgery, The Sixth Affiliated Hospital of Sun Yat-sen University, 510655 Guangzhou, China; 8 Zhongshan School of Medicine, Sun Yat-sen University, 510080 Guangzhou, China; 9 Instrumental Analysis & Research Center, Sun Yat-sen University, 510080 Guangzhou, China; 10 Department of Colorectal Surgery, The First Affiliated Hospital of Sun Yat-sen University, 510000 Guangzhou, China; 11 Metabolomics Core Facility, Center for Cancer Biology, VIB, Leuven, Belgium

**Keywords:** Cancer metabolism, Ubiquitylation, Colorectal cancer

## Abstract

The Serine–Glycine–One-Carbon (SGOC) pathway is pivotal in multiple anabolic processes. Expression levels of SGOC genes are deregulated under tumorigenic conditions, suggesting participation of oncogenes in deregulating the SGOC biosynthetic pathway. However, the underlying mechanism remains elusive. Here, we identified that Interleukin enhancer-binding factor 3 (ILF3) is overexpressed in primary CRC patient specimens and correlates with poor prognosis. ILF3 is critical in regulating the SGOC pathway by directly regulating the mRNA stability of SGOC genes, thereby increasing SGOC genes expression and facilitating tumor growth. Mechanistic studies showed that the EGF–MEK–ERK pathway mediates ILF3 phosphorylation, which hinders E3 ligase speckle-type POZ protein (SPOP)-mediated poly-ubiquitination and degradation of ILF3. Significantly, combination of SGOC inhibitor and the anti-EGFR monoclonal antibody cetuximab can hinder the growth of patient-derived xenografts that sustain high ERK-ILF3 levels. Taken together, deregulation of ILF3 via the EGF–ERK signaling plays an important role in systemic serine metabolic reprogramming and confers a predilection toward CRC development. Our findings indicate that clinical evaluation of SGOC inhibitor is warranted for CRC patients with ILF3 overexpression.

## Introduction

Colorectal cancer (CRC), including the carcinogenesis of colon and rectal, is an important contributor to cancer mortality and morbidity.^1,2^ Thus, there is an urgent need to identify risk factors, biomarkers, and effective treatment strategies for CRC. The monoclonal antibody cetuximab inhibits EGFR by targeting extracellular domain and has clinically meaningful activity in terms of disease remission and survival in metastatic CRC. However, only a subgroup of patients treated with cetuximab benefits from the drug.^3^ The detailed picture of downstream pathways deregulated in EGFR signaling remains unclear and needs to be elucidated to understand cancer pathogenesis and develop efficient treatments.^4^

Deregulation of cellular energetics (metabolic reprogramming), one of the cancer hallmarks, involves tumor cells to rewire metabolic pathways to support rapid proliferation, continuous growth, metastasis, and resistance to therapies.^5,6^ Serine biosynthesis supports several metabolic processes that are crucial for the growth and survival of proliferating cells.^7–9^ For examples, loss of LKB1 tumor suppressor leads to activation of serine biosynthesis to support tumor growth and DNA methylation.^10^ K-ras-driven pancreatic and intestinal cancers are less responsive to serine/glycine depletion-mediated cancer inhibition.^11^ Genes involved in the serine synthesis pathway, including *PHGDH*, *PSAT1*, *PSPH*, *SHMT1* and *SHMT2*, are deregulated in several cancers. These genes encode enzymes to produce serine and glycine from glycolytic intermediate 3-phosphoglycerate. Phosphoglycerate dehydrogenase (PHGDH), the first enzyme of the SGOC pathway, is amplified in breast cancer and melanomas.^12,13^ NRF2 and Myc activate the expression of genes in serine synthesis pathway in cancer.^14,15^ HER2 and p53 regulate the expression of PHGDH.^9^ Thus, serine biosynthesis pathway is regulated by many oncogenes and tumor suppressor genes. However, the regulation of cancer serine biosynthesis is not fully understood, suggesting that more regulators remain to be identified.

Serine is a critical one-carbon unit donor involved in methionine cycle and folate cycle, contributing to nucleotide synthesis, methylation reactions and the generation of NADPH for antioxidant defense.^10,16^ Cancer cells particularly utilize serine as a major source of one-carbon units, so they rely on extracellular serine uptake or SGOC for accelerating cell growth and proliferation.^11,13^ Despite the significant roles of serine in many cellular processes, the regulatory mechanism behind serine metabolism in cancer is not fully understood, and the link of serine metabolic/mechanistic deregulation and clinical aggressiveness is not well characterized. Understanding the importance of serine metabolism in cancer is beginning to provide new opportunities for therapeutic intervention, while the ability to selectively target serine synthesis remains elusive.

ILF3, also known as NF90/NF110, encodes a double-stranded RNA (dsRNA)-binding protein that complexes with other proteins, mRNAs, small noncoding RNAs, and dsRNAs to regulate gene expression and stabilize mRNAs.^17,18^ Through binding to different cellular RNAs, ILF3 participates in diverse cellular functions such as mRNA stabilization, translation inhibition, and noncoding RNA biogenesis.^19^ The role of ILF3 in cancer biology is emerging, and it was found to regulate vascular endothelial growth factor mRNA stability in breast cancer as well as to regulate the cell cycle of hepatocellular carcinoma cells through modulation of cyclin E1 mRNA stability.^20,21^ However, much of its activity in cancer remains uncharacterized. The cause and consequence of ILF3 abundance in cancer are not well elucidated. Furthermore, the role of ILF3 in cancer hallmark deregulation, such as metabolism reprogramming, has never been reported.

In this study, we show that the frequent overexpression of ILF3 in CRC results in the metabolic reprogramming phenotype in serine biosynthesis that promotes tumor growth, organoid formation, and correlates with poor cancer survival. We demonstrate that the EGFR–ERK axis accelerates tumor-promoting metabolic programs by mitigating SPOP-mediated ILF3 poly-ubiquitination and subsequent degradation. Therefore, EGFR signaling leads to ILF3 stabilization, promotes the mRNA stability of SGOC genes, and furthers serine biogenesis in cancer. Our study uncovers ILF3 as an important regulator of serine–glycine metabolic pathway and holds the potential to provide more effective cancer treatment strategies.

## Results

### ILF3 is overexpressed in CRC and is a prognostic marker correlated with poor survival

**Fig. 1:**
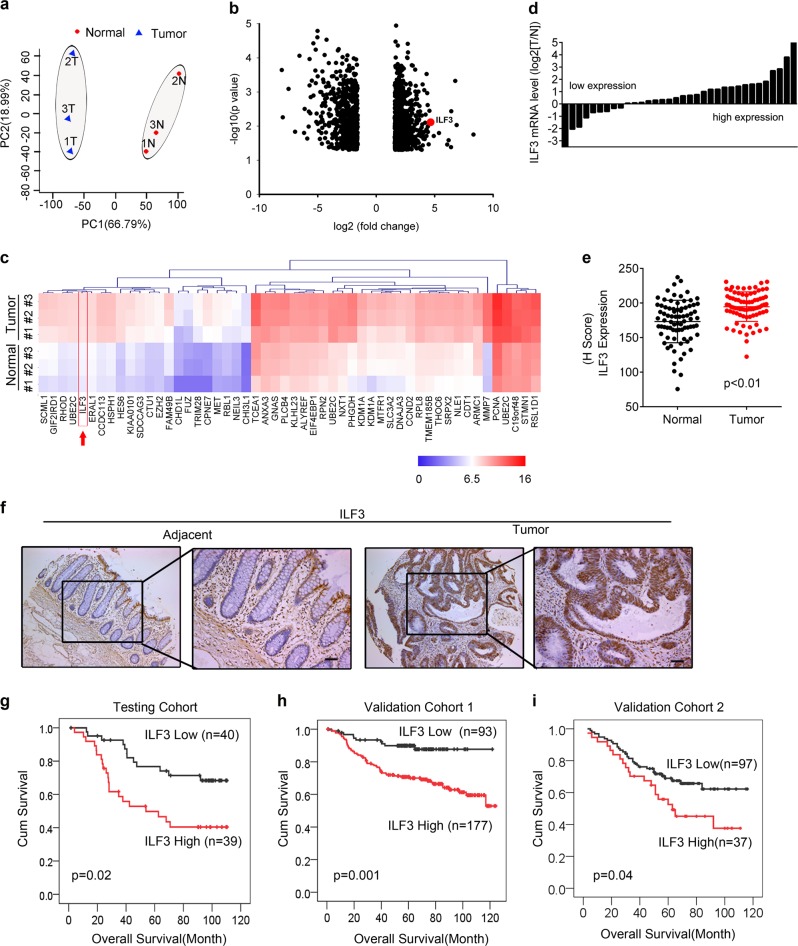
Identification of ILF3 as a poor prognosis marker in CRC. **a** Principal component analysis of microarrays from three paired CRC and normal tissue samples. PC1 and PC2 explain 66.79% and 18.99% of the variation, respectively. **b** Volcano plot analysis of different gene expressions after knockdown of ILF3 (*P* < 0.05, ∣fold change∣ > 3). **c** Heatmap of the top 50 genes that were upregulated in CRC. The color scale indicates a log_2_ ratio of the normalized hybridization signal intensities of the upregulated genes. **d** Waterfall plot of the relative ILF3 mRNA levels from 34 paired samples of CRC and normal tissue measured using qRT-PCR. **e** Expression of ILF3 in 79 paired normal and CRC tissue samples. **f** Representative images of ILF3 IHC staining in human colon cancer and adjacent normal colon tissue. Scale bars, 50 μm. **g**–**i** Kaplan–Meier survival curves based on ILF3 expression in the CRC tissues of the testing and validation cohorts. The receiver operating characteristic curve was used to define the cutoff, and log-rank analysis was used to test for significance.

To identify potential deregulated genes in CRC tumorigenesis, we examined the microarray gene expression profile of colorectal cancer tissues from patients with newly diagnosed CRC versus adjacent normal tissues. A comparison of the gene expression changes in CRC patients indicated that ILF3 was among the top 50 genes upregulated in tumor cells when compared with adjacent normal cells from CRC patients (Fig. 1a–c). Further analysis of 34 paired samples of colon cancer tissue and adjacent normal mucosa again and various cell lines revealed that CRC exhibits high ILF3 levels (Fig. 1d; Supplementary information, Fig. S1a). In addition, analysis of the mRNA expression data from the colon cancer set GSE9348 and The Cancer Genome Atlas (TCGA) demonstrated that the ILF3 expression level was higher in cancer tissue than in normal tissue (Supplementary information, Fig. S1b and c). Notably, ILF3 expression is also elevated in many types of human tumors in addition to CRC (Supplementary information, Fig. S1d). Immunohistochemical staining of ILF3 in tissue microarrays (*n* = 79) revealed that ILF3 expression in CRC is higher than normal tissue (Fig. 1e, f). Kaplan–Meier analysis showed that high ILF3 levels were correlated with poor overall survival in three independent cohorts (Fig. 1g–i). The testing cohort included paired CRC and normal colon tissue samples from 79 patients. The validation cohorts included CRC samples from 270 patients and 134 patients, respectively. High ILF3 expression was positively correlated with advanced clinical stage (Supplementary information, Table S1). Furthermore, multivariate Cox regression analysis revealed that ILF3 expression was an independent prognostic factor for poor survival (Supplementary information, Table S2).

ILF3 knockdown via siRNA transfection had a marked negative effect on cell proliferation (Supplementary information, Fig. S2a). Immunoblot analysis of endogenous ILF3 in six CRC cell lines revealed that ILF3 expression was relatively high in HCT-8, DLD-1 and HCT-116 cells (Supplementary information, Fig. S2b). Introduction of short-hairpin RNA (shRNA) to knockdown ILF3 inhibited the cell proliferation and sphere formation ability of these colorectal cancer cells (Supplementary information, Fig. S2c–e). In conclusion, ILF3 is highly expressed in CRC and is correlated with poor survival, and its positive impacts on cell growth and sphere formation contribute to its oncogenic role.

### ILF3 reprograms serine metabolism to maintain CRC malignant progression

**Fig. 2:**
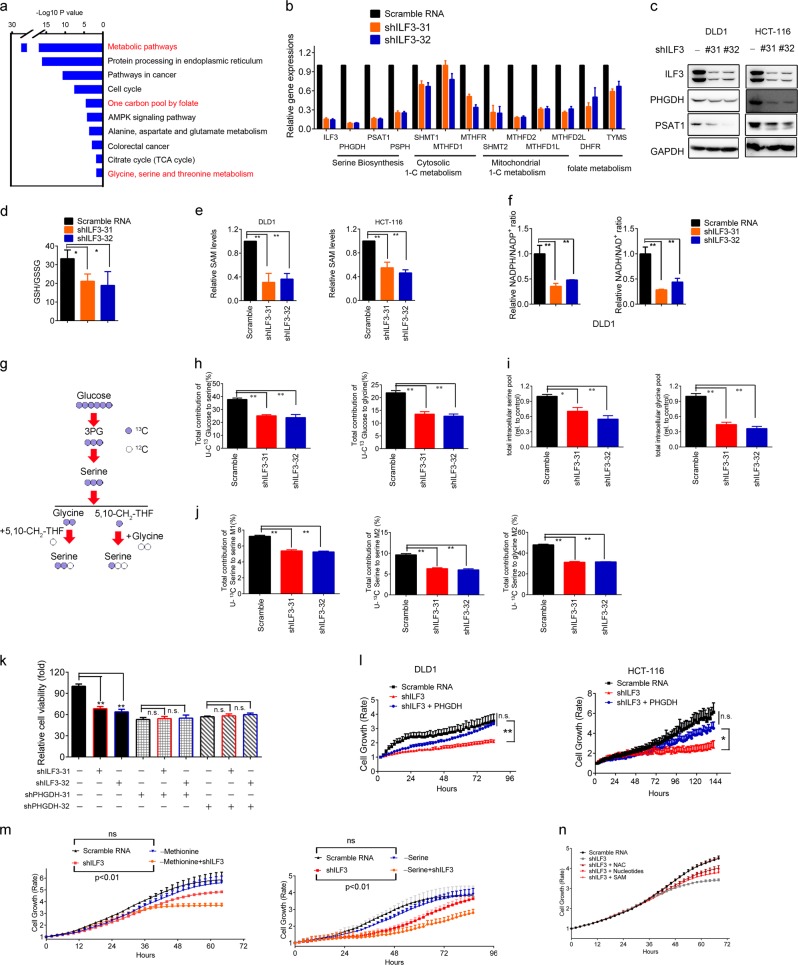
Activation of de novo serine biosynthesis in ILF3-dependent CRC. **a** Gene categories significantly (*P* *≤* 0.05) enriched for genes deregulated in metabolism, owing to knockdown of ILF3 in the CRC cell line DLD1. **b** Gene abundance of the serine biosynthesis pathway after knockdown of ILF3 using shRNA. The data are presented as the means ± SD. **c** Immunoblot analysis of serine synthesis pathway proteins (PHGDH and PSAT1) after knockdown of ILF3 using shRNA. **d** Relative ratios of reduced to oxidized glutathione (GSH/GSSG) in cell lines determined by LC–MS/MS. **e** Measurement of SAM in DLD1 and HCT-116 cells transduced with ILF3 shRNA. **f** Measurement of NADPH/NADP^+^ and NADH/NAD^+^ levels in DLD1 cells with or without ILF3 KD. **g** Schematic metabolic map of [U-^13^C-labeled serine metabolism. **h** Incorporation of carbon-13 (^13^C) from [U-^13^C] glucose (11 mM) into the indicated metabolites at 24 h in DLD1 cells. The data are presented as the means ± SD. **i** Intracellular pool levels of serine and glycine at 24 h in DLD1 cells. **j** Incorporation of carbon-13 (^13^C) from [U-^13^C] serine (0.4 mM) into the indicated metabolites at 24 h in DLD1 cells. The data are presented as the means ± SD. **k** Relative cell growth rate of DLD1 cells transduced with the indicated constructs. **l** Relative cell growth rate of DLD1 and HCT-116 cells transduced with the indicated constructs. **m** Relative cell growth rate of DLD1 and HCT-116 cells cultured with or without serine or methionine. The data are presented as the means ± SD. **n** Relative cell growth rate of DLD1 cells cultured with or without NAC, nucleotides or SAM upon ILF3 KD. The data are presented as the means ± SD.

To further identify the roles of ILF3 in colorectal cancer, we performed transcriptomic analysis in ILF3-knockdown (KD) DLD1 cells. Notably, gene ontology analysis data indicated that there are significant differences between the ILF3-KD cells and control cells in multiple metabolism-related pathways, including networks that connect glycolytic intermediates to serine–glycine one-carbon cycle (SGOC) metabolism (Fig. 2a).^22^ For example, folate metabolism and the serine–glycine pathway scored high among the induced pathways. We then performed gene set enrichment analysis (GSEA) to figure out the association between expression of ILF3 and signaling pathways in two sets of colon cancer GSE5206 and GSE17538. We found that ILF3 was positively correlated with KEGG one-carbon folate pathway (Supplementary information, Fig. S3a and b). Furthermore, there was an enrichment of a 64-gene signature defining the entire SGOC network in the presence of high ILF3 in GSEA of the colon cancer set GSE29621, indicating coordinated activation of the network by ILF3 (Supplementary information, Fig. S3c).^23^

SGOC metabolic pathway deregulation plays a role in cancers.^15^ We verified ILF3’s impact on SGOC and confirmed that ILF3 KD led to reduced expression of genes (*PHGDH*, *PSAT1*, *PSPH*, *SHMT1*, *SHMT2*, etc.) involved in the SGOC pathway (Fig. 2b, c). Serine metabolism can also fuel the redox homeostasis and methionine salvage pathway, which leads to production of the methyl donor S-adenosyl methionine (SAM) to control metabolic and epigenetic homoeostasis. We showed that SAM levels, GSH/GSSG ratios, NADPH/NADP^+^ ratios and NADH/NAD^+^ ratios were reduced in ILF3-KD cells (Fig. 2d–f). Significantly, applying uniformly carbon-13-labeled glucose ([U-^13^C] glucose) in metabolite tracing demonstrated that ILF3 KD in DLD1 cells leads to a pronounced decrease in the serine and glycine biosynthesis rates and serine and glycine pools in CRC cells (Fig. 2g–i; Supplementary information, S3d). To determine the fate of exogenous serine, we fed DLD1 cells [U-^13^C] serine instead of unlabeled serine in the medium. ILF3 KD reduced the production of M + 2-Glycine, M + 2 serine (arose from glycine and unlabeled 5,10-^13^CH_2_-THF) and M + 1 serine (arose from unlabeled glycine and 5,10-^13^CH_2_-THF) (Fig. 2j). PHGDH KD leads to reduced cell viability. Notably, cells with PHGDH KD showed no significant decrease in viability in addition to ILF3 KD, and PHGDH overexpression rescued the cell viability inhibition by ILF3 KD (Fig. 2k, l; Supplementary information, Fig. S3e). Also, cells expressing high levels of ILF3 showed no significant decrease in proliferation after culture in serine or methionine-depleted medium (Fig. 2m). The cell growth rate under both ILF3 KD and serine- or methionine-free conditions was slower than that with ILF3 KD alone, suggesting that ILF3-high cells are less dependent on an exogenous supply of the amino acid. Methionine and one-carbon metabolism (especially SAM) are critical for tumor-initiating cells.^24–26^ SAM, N-Acetylcysteine (NAC) and nucleotides also rescued the decreased cell viability caused by ILF3 KD (Fig. 2n).

Glycolytic intermediates are known to be critical for SGOC.^27^ It has been shown that serine is an allosteric activator of pyruvate kinase isoform 2 (PKM2), which is involved in the last step of glycolysis.^28^ Interestingly, we showed that the intracellular lactate dehydrogenase (LDH) activity was decreased in ILF3-KD cells (Supplementary information, Fig. S3f). Additionally, ILF3 KD in DLD1 and HCT-116 cells led to a decreased oxygen consumption rate (OCR) and extracellular acidification rate (ECAR) (Supplementary information, Fig. S3g), suggesting that ILF3 plays a role in crosstalk between SGOC and glycolysis.

In the canonical pathway of glucose-derived serine synthesis, PHGDH catalyzes the first, rate-limiting step of the SGOC pathway by shunting 3-phosphoglycerate to the SGOC pathway. With quantitative high-throughput screening, a previous report identified a new small-molecule PHGDH inhibitor, NCT-503.^29^ We performed cell viability assays and found that NCT-503 inhibited the growth of ILF3-high CRC cell lines in a dose-dependent manner, while ILF3-low CRC cells were less responsive (Supplementary information, Fig. S4a). Basically, ILF3 expression levels were negatively correlated with the IC50 values of NCT-503 (Supplementary information, Fig. S4b). Thus, ILF3 KD in ILF3-high cells led to an increase in the NCT-503 IC50. NCT-503i, the inactive control probe of NCT-503, did not have the similar impacts in the experiments (Supplementary information, Fig. S4c and d). In conclusion, CRC with ILF3 overexpression is more dependent on the increased activity of the serine biosynthesis pathway to support tumor growth.

### ILF3 Interacts with and stabilizes the mRNA of SGOC genes to boost gene expression

**Fig. 3:**
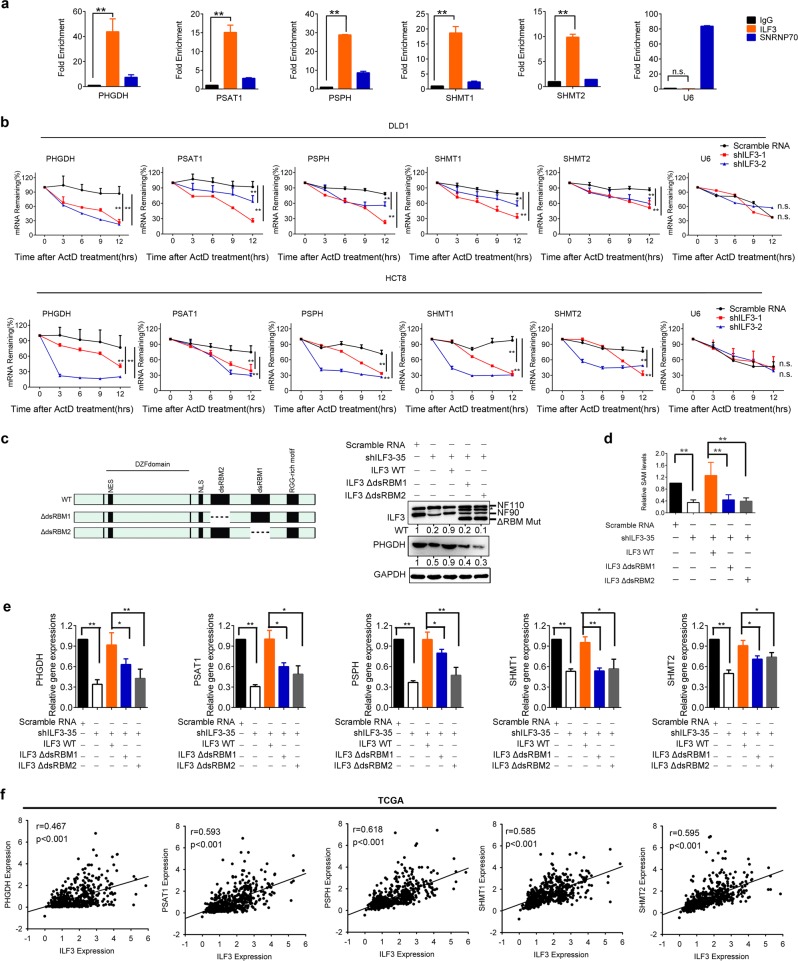
ILF3 directly regulates SGOC gene mRNA stability. **a** ILF3 binds to the mRNAs of SGOC pathway genes. RNA immunoprecipitation (RIP) was performed in DLD1 cells using anti-ILF3, anti-SNRNP70 (control) or anti-IgG antibodies, followed by RT-qPCR with primers recognizing the indicated mRNAs. The fold expression of RIP-enriched mRNAs relative to the input was calculated. The data are presented as the means ± SD. **b** RT-PCR of serine pathway genes in DLD1 and HCT-8 cells transduced with ILF3 shRNA followed by actinomycin D treatment (10 μg/mL) over time. The data are presented as the means ± SD. **c** A schematic drawing of ILF3 RBM-truncated mutants and their expression. Asterisk indicates non-specific band. **d** The decreased production of SAM in ILF3 KD cells could be rescued by reintroduction of WT ILF3 but not the ILF3 dsRBM mutants. The data are presented as the means ± SD. **P < 0.01. **e** The decreased gene expression levels of *PHGDH*, *PSAT1*, *PSPH*, *SHMT1* and *SHMT2* in ILF3-KD cells could be rescued by reintroduction of ILF3 WT but not RBM-truncated mutants. The data are presented as the means ± SD. *P *<* 0.05; **P < 0.01. **f** Correlation analyses of ILF3 and SGOC genes in 521 CRC patients. The human CRC patient dataset was obtained from TCGA.

Although ILF3 has dsRNA-binding motifs (dsRBMs) and is an RNA-binding protein (RBP),^21,30^ its mRNA targets in cancer remain largely unknown.^31^ To further analyze the role of ILF3 in regulating SGOC genes, we performed RNA immunoprecipitation followed by qRT-PCR and found that ILF3 interacts with the mRNA of SGOC genes. Quantitation of the binding between ILF3 and SGOC gene mRNAs revealed that the binding of ILF3 to PHGDH, PSAT1, PSPH, SHMT1 and SHMT2 mRNAs was at least 10-fold enriched compared with that of immunoglobulin G (IgG) and anti-SNRNP70 (Fig. 3a). We also included U6 RNA as a control to indicate the specificity of the binding (Fig. 3a). Because ILF3 is involved in mRNA turnover and in the regulation of cellular translation,^19,32–34^ we hypothesized that these mRNA/ILF3 interactions might play a role in regulating SGOC mRNA stability. Indeed, blocking de novo transcription with actinomycin D in CRC cells transfected with ILF3 shRNA led to increased mRNA turnover of SGOC genes when compared with cells transfected with the scrambled control (Fig. 3b). U6 RNA served as a control and its turnover was not affected by ILF3 shRNA (Fig. 3b). Together, these results clearly indicate that specific binding of ILF3 to mRNAs stabilized targeted SGOC gene mRNAs to promote gene expression.

ILF3 selectively binds mRNAs or circRNAs through the dsRBM.^31,35^ To verify the function of the ILF3 dsRBM in regulating SGOC gene stability and expression, we reintroduced short hairpin (sh)RNA-resistant human wild-type (WT) ILF3 or mutants lacking the dsRBM into cells with ILF3 KD to examine the SGOC gene expression levels. We found that reintroducing WT Flag-ILF3 could rescue SGOC gene expression and SAM levels, while reintroducing ILF3 dsRBM-deleted mutants attenuated the rescue capability (Fig. 3c–e). Moreover, we searched TCGA database for the expression profiles of ILF3 and SGOC-related genes and found that transcriptomic analysis of 521 colon cancer samples showed a positive correlation between ILF3 and SGOC-related gene expression (Fig. 3f). The expression correlation translates into clinical significance in terms of survival. Kaplan–Meier survival analysis showed a significantly shorter overall survival in colorectal adenocarcinoma patients with high ILF3, PHGDH, or SHMT2 gene expression (Supplementary information, Fig. S4e). Hence, ILF3 directly interacts with the mRNA of SGOC genes and is a positive regulator of the SGOC pathway, promoting CRC development and resulting in poor survival.

### The EGF-ERK axis regulates ILF3 stability and the SGOC network

**Fig. 4:**
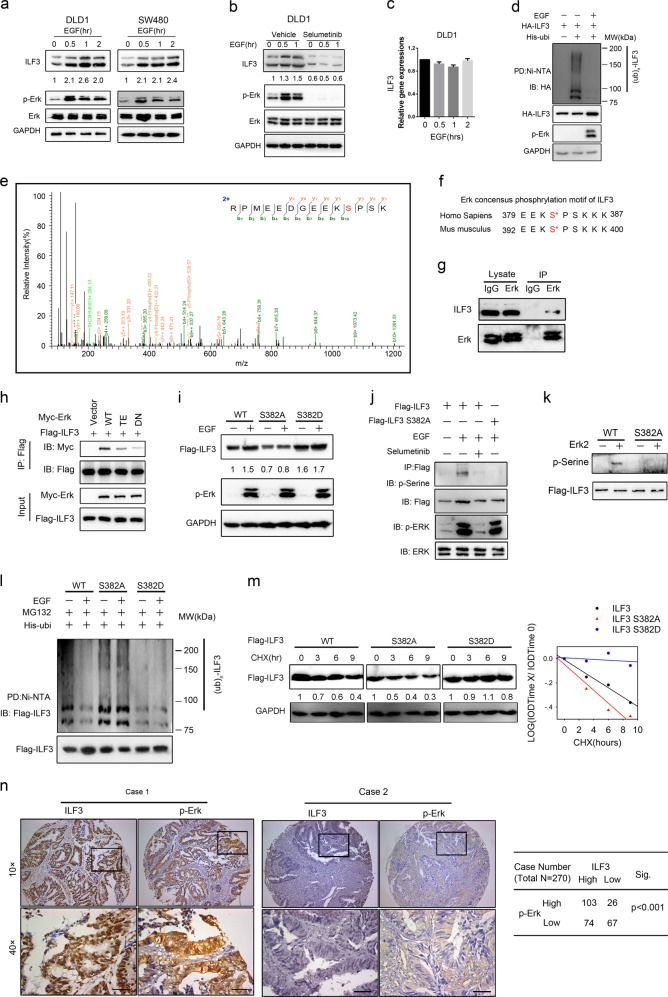
The EGF-ERK axis regulates ILF3 stability and the SGOC network. **a** Immunoblot analysis of ILF3 protein in cells serum-starved and stimulated with EGF (100 ng/mL). **b** Immunoblot analysis of ILF3 protein in cells treated with EGF (100 ng/mL) and selumetinib (2 μM). **c** ILF3 mRNA expression in cells treated with EGF (100 ng/mL). **d** Immunoblot analysis of ubiquitinated ILF3 protein in DLD1 cells treated with or without EGF (100 ng/mL). MG132 (20 μM) was added to the cells 6 h before they were harvested with guanidine-HCl-containing buffer. The cell lysates were pulled down with nickel beads and immunoblotted with an anti-ILF3 antibody. **e** MS/MS of the ILF3 S382 phosphorylation site. The fragmentation spectrum of the typical ILF3 peptide RPMEEDGEEKS < phos > PSK (566.9075*m*/*z*, 3+) carrying the modification is shown with annotated y-ions (orange) and b-ions (green). The y-ion series covers the phosphorylation site and displays the typical neutral addition of 79.9663 Da (corresponding to the addition of PO_3_). **f** Erk consensus phosphorylation motif of ILF3. **g** Immunoblot analysis of the indicated proteins from immunoprecipitates (IP) obtained from 293T cells with MG132 treatment for 6 h. **h** Immunoblot analysis of samples from co-IP with anti-Flag antibody in 293T cells transfected with the indicated constructs and treated with 20 µM MG-132 for 6 h. **i** Immunoblot analysis of ILF3 mutant in 293T cells transfected with the indicated constructs in the presence or absence of EGF. **j** Immunoblot analysis of samples from co-IP with anti-Flag antibody in 293T cells transfected with the indicated constructs and treated with 20 µM MG-132 for 6 h. **k** Immunoblot analysis of samples from in vitro kinase assay of Erk2. **l** Immunoblot analysis of poly-ubiquitinated ILF3 in poly-ubiquitination assays of 293T cells transfected with the indicated constructs and treated with 20 µM MG132 for 6 h. EV, empty vector. **m** Immunoblot analysis of the ILF3 protein turnover rate in 293T cells treated with cycloheximide (CHX). **n** Correlation of ILF3, and p-ERK staining in human 270 CRC tissue microarray samples. Representative images are shown.

To further understand the upstream regulation of ILF3, we next determined whether the activation of EGFR, which is overexpressed or mutated in many cancer types, including CRC, has an effect on ILF3 expression. EGF can increase phosphorylation level of ERK independently from KRAS status.^36,37^ We found that EGF treatment in DLD1 cells activated ERK and increased the steady-state expression of ILF3 without affecting its mRNA levels (Fig. 4a–c). Moreover, EGF treatment decreased the poly-ubiquitination level of ILF3 (Fig. 4d; Supplementary information, Fig. S5a) and decelerated the turnover rate of ILF3 in the presence of the de novo protein synthesis inhibitor cycloheximide (Supplementary information, Fig. S5b). Mass spectrometry analysis identified phosphorylated serine residue in ILF3 at the S382, showing a characteristic tandem mass spectrometry (MS/MS) spectrum including C-terminal y-ions and amino-terminal b-ions (Fig. 4e). Sequence analysis of ILF3 using the NetPhos algorithm (http://www.cbs.dtu.dk/services/NetPhos) revealed that ILF3 contains an ERK consensus phosphorylation motif (Ser-Pro) at the S382 residues (Fig. 4f). A coimmunoprecipitation (co-IP) assay demonstrated that ERK1/2 binds to ILF3 (Fig. 4g). MAPKs bind to their substrates through a docking groove comprised of an acidic common docking (CD) domain and a glutamic acid-aspartic acid pocket. Mutations in the CD domain (D318/321N) or the glutamic acid–aspartic acid pocket (T159/160E) reduced the binding of Erk2 with ILF3 (Fig. 4h). Next, we constructed S382A and S382D site mutants of ILF3. The S382A mutant did not respond to EGF-induced upregulation compared with WT. The phosphor-mimetic S382D showed a higher protein level even in the absence of EGF (Fig. 4i). Moreover, we performed phos-Serine detection on immunoprecipitate of ILF3, and found that EGF treatment led to Serine phosphorylation of ILF3 but not on ILF3 S382A. Consistently, MEK inhibitor selumetinib abolished EGF-mediated serine phosphorylation on ILF3 (Fig. 4j). In an in vitro kinase assay, we observed that Erk2 catalyzed serine phosphorylation on WT ILF3 but not on S382A mutant (Fig. 4k). To characterize the significance of this S382 phosphorylation, we performed ubiquitination experiment and found that while EGF treatment reduced the poly-ubiquitination level of WT ILF3, it had little effect on the poly-ubiquitination level of ILF3 S382A mutant (Fig. 4l). The phosphor-mimetic S382D mutant was resistant to being poly-ubiquitinated (Fig. 4l). Also, ILF3 S382A mutant had a fast turnover rate when compared with WT ILF3, whereas the turnover rate of the phospho-mimetics ILF3 S382D mutant was slow (Fig. 4m). Significantly, p-Erk2 expression positively correlates with ILF3 in CRC as analyzed in 270 CRC tissue microarrays (Fig. 4n). Together, ERK2-mediated ILF3 phosphorylation on S382 leads to reduced poly-ubiquitination and increased stability of ILF3.

We then observed that inhibition of the ERK signaling with selumetinib, a selective ERK inhibitor, also inhibited SGOC pathway genes and metabolites, suggesting that ERK works on ILF3 to regulate the SGOC pathway (Supplementary information, Fig. S5c and d). Moreover, overexpression of ILF3 recovered the SAM levels inhibited by selumetinib (Supplementary information, Fig. S5e). These data indicate that EGF-ERK activity affects SGOC activation via regulating the expression level of ILF3.

### SPOP is the E3 ligase that regulates ILF3 protein stability

**Fig. 5:**
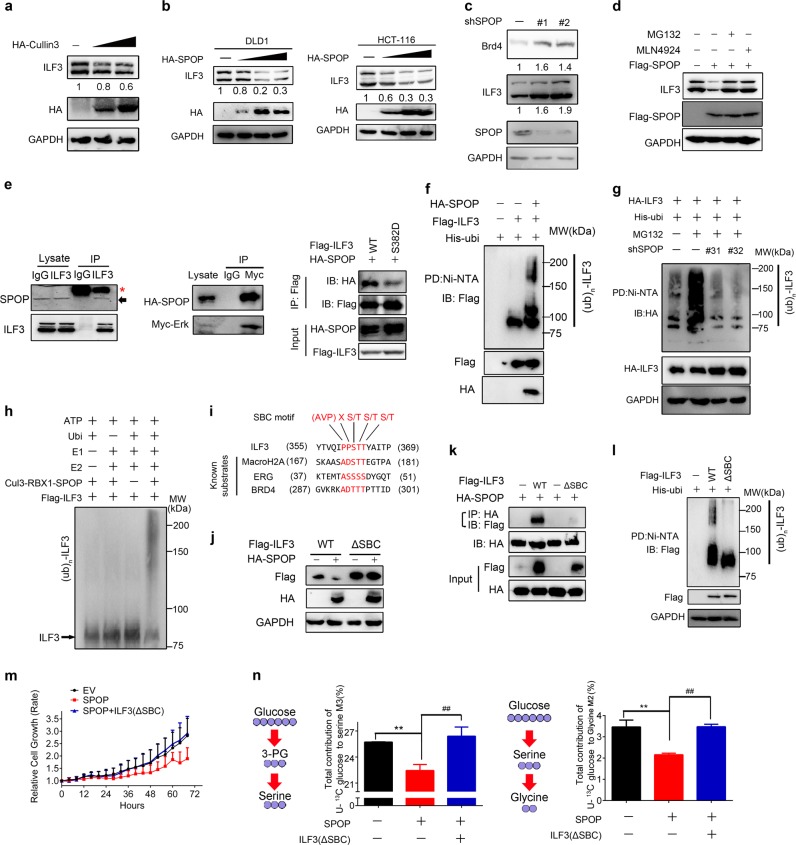
SPOP is the E3 ligase that regulates ILF3 protein stability. **a** Immunoblot analysis of ILF3 from 293T cells transfected with the HA-Cullin3 plasmid. **b** Immunoblot analysis of ILF3 from DLD1 and HCT-116 cells transfected with the HA-SPOP plasmid. **c** Immunoblot analysis of ILF3 from 293T cells transfected with the shSPOP plasmid. **d** Immunoblot analysis of ILF3 from 293T cells transfected with the Flag-SPOP plasmid in the presence or absence of MG132 or MLN4924. **e** Immunoblot analysis of the indicated proteins from immunoprecipitates (IP) obtained from 293T cells with MG132 treatment for 6 h. Asterisk, heavy chain. The arrow indicates endogenous SPOP. **f**, **g** Immunoblot analysis of poly-ubiquitinated ILF3. Cells were transfected with the indicated constructs and then treated with MG132 for 8 h. The cell lysates were pulled down with nickel beads and immunoblotted with indicated antibody. **h** Immunoblot of ILF3 in vitro ubiquitination assay by the CUL3–RBX1–SPOP E3 ligase complex. Affinity-purified SPOP complexes were incubated with purified Flag-ILF3 protein, ATP, E1 and E2 enzymes, and his-ubiquitin. **i** Amino acid sequence alignment of the putative substrate-binding consensus (SBC) motif in ILF3. MacroH2A, ERG and BRD4 are known SPOP substrates containing well-characterized SBC motifs. The amino acids of SBC motifs among the indicated proteins are depicted in red. (AVP) refers to the allowed amino acid groups. (X) denotes any type of residue. **j** Immunoblot analysis of protein levels of ILF3 constructs from cells transfected with HA-SPOP. **k** Immunoblot analysis of samples from co-IP with anti-HA antibody in 293T cells transfected with the indicated constructs and treated with 20 µM MG-132 for 6 h. **l** Immunoblot analysis of poly-ubiquitinated ILF3 in poly-ubiquitination assays of 293T cells transfected with the indicated constructs and treated with 20 µM MG132 for 6 h. EV, empty vector. **m** Cells were transfected with the indicated constructs and cell proliferation rates were measured. The data are presented as the means ± SD. **n** Incorporation of carbon-13 (^13^C) from U[^13^C] glucose (11 mM) into the indicated metabolites at 24 h in DLD1 cells. The data are presented as the means ± SD. **P *<* 0.01; ##*P* *<* 0.01.

To identify the E3 ligase that governs ILF3 protein turnover, we performed proteomics analysis and identified that Cul3 could interact with ILF3. The Cul3-based E3 ligases include Keap1 and SPOP. However, we showed that SPOP but not Keap1 could reduce ILF3 protein expression (Fig. 5a, b; Supplementary information, Fig. S6a), and SPOP KD increased ILF3 protein expression (Fig. 5c). To understand how SPOP regulates ILF3 levels, we treated cells with proteasome inhibitor MG132, or with cullin-based E3 ligase inhibitor MLN4924 and found increased ILF3 protein levels (Fig. 5d). We performed co-IP experiments and detected the interaction of SPOP–ILF3 and SPOP–ERK (Fig. 5e; Supplementary information, Fig. S6b), suggesting the existence of a ternary complex. However, ILF3 S382D mutation decreases its interaction with SPOP (Fig. 5e), suggesting that ERK2-mediated phosphorylation is involved in the regulation of the binding process. This interaction of SPOP–ILF3 is significant because SPOP overexpression resulted in reduced ILF3 steady-state expression, facilitated ILF3 poly-ubiquitination and accelerated ILF3 protein turnover (Fig. 5f, g; Supplementary information, Fig. S6c). Furthermore, we found that the SPOP–CUL3–RBX1 complex catalyzed ILF3 ubiquitination in vitro (Fig. 5h). We performed sequence scanning on the ILF3 amino acid sequence and found that one evolutionarily conserved putative E3 ligase SPOP-binding consensus (SBC) motif [AVP]X[ST][ST][ST] is present in ILF3,^38^ suggesting that ILF3 is a potential degradation target of the E3 ligase SPOP (Fig. 5i; Supplementary information, Fig. S6d). On the other hand, the ILF3 ΔSBC mutant with the SBC motif deleted was not responsive to the SPOP-mediated reduction in steady-state expression, failed to interact with SPOP, was resistant to SPOP-mediated poly-ubiquitination, and manifested a slower turnover rate (Fig. 5j–l; Supplementary information, Fig. S6e). Additionally, the ILF3 ΔSBC mutant rescued the cell viability, serine and glycine metabolism inhibited by SPOP overexpression (Fig. 5m, n).Thus, SPOP efficiently mediates the ubiquitin-mediated protein degradation of ILF3 through physical interaction. Significantly, low SPOP expression correlates with high ILF3 in CRC as analyzed in 270 CRC tissue microarrays (Supplementary information, Fig. S6f), recapitulating the regulation between SPOP and ILF3 in cell line studies.

### Cancer type-specific SPOP mutants fail to regulate ILF3 protein stability

SPOP plays a potential role in tumorigenicity because it is a frequently mutated gene in primary cancer.^39–45^ SPOP is a member of the MATH–BTB protein family, containing an N-terminal meprin and TRAF homology (MATH) domain (responsible for substrate recognition and interaction) and a C-terminal BTB domain (binds CUL3 and forms the functional E3 ubiquitin ligase complex) (Supplementary information, Fig. S7a).^46^ BTB domain-deleted SPOP lost its effect on mediating ILF3 poly-ubiquitination (Supplementary information, Fig. S7b). We found that several CRC-derived SPOP mutations clustered within the MATH domain, and determined that these CRC-associated SPOP mutants (including R70Q, R138C and L142I) failed to regulate and interact with ILF3 (Supplementary information, Fig. S7c and d); therefore, they could not promote ILF3 poly-ubiquitination (Supplementary information, Fig. S7e). Notably, these mutations are all located at the substrate-binding middle groove of the SPOP MATH domain based on structure modeling (Supplementary information, Fig. S7f),^46^ implying that structural change leads to compromised substrate-binding activity. Additionally, these mutants failed to reduce the half-life of ILF3 (Supplementary information, Fig. S7g), thereby attenuating their impacts on reducing SGOC gene expression and SAM levels when compared with the effects of WT SPOP (Supplementary information, Fig. S7h and i). Together, our data show that cancer-derived SPOP mutants fail to interact with ILF3, thereby leading to ILF3 stabilization and subsequent SGOC gene activation.

### Knockdown of ILF3 or inhibition of serine biosynthesis mitigates tumor growth in vivo

**Fig. 6:**
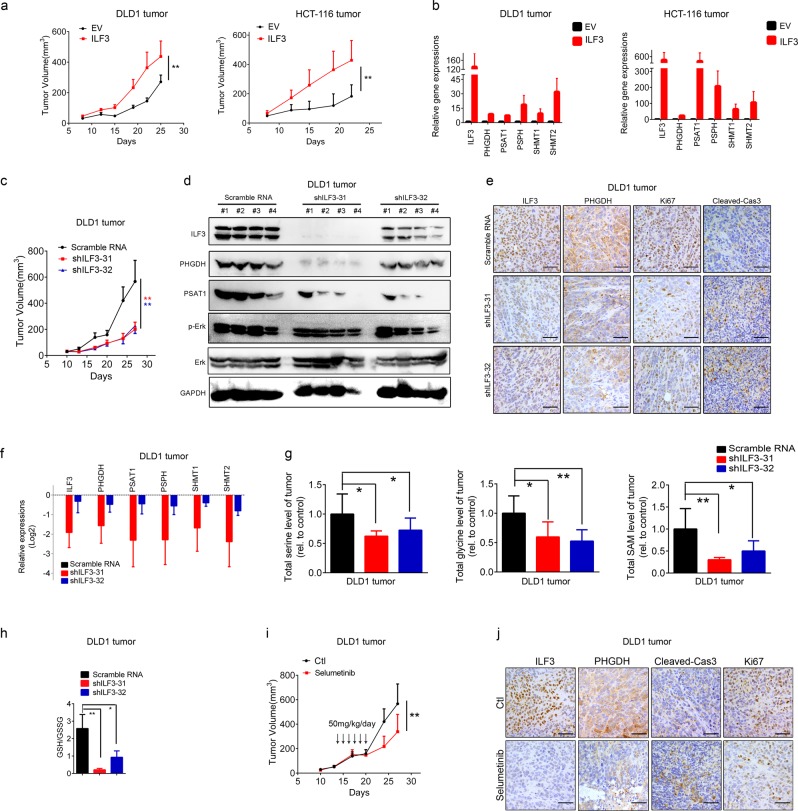
ILF3 promotes tumor growth in vivo. **a** Tumor growth curves of DLD1 (1 × 10^6^) or HCT-116 (1 × 10^6^) colon cancer cells with or without ILF3 overexpression. Cells were subcutaneously injected into nude mice (*n* = 6). The tumors were isolated at the end of the experiments. **b** Serine pathway gene expression in the tumor tissues of (**a**). qRT-PCR analysis was performed to measure the mRNA levels of serine biosynthesis pathway genes. **c** Measurement of the subcutaneous tumor growth of ILF3-KD DLD1 cells (1 × 10^7^). *n* *=* 9/group. The data are presented as the means ± SD. **d** Immunoblot analysis of protein levels of ILF3, p-Erk, Erk, PHGDH and PSAT1 in the subcutaneous tumor tissues generated in (**c**). **e** Representative IHC images of ILF3, PHGDH, Ki-67 and cleaved-Caspase-3 staining in the subcutaneous tumor tissues generated in (**c**). Scale bars represent 50 μm. **f** SGOC pathway gene expression after ILF3 KD in subcutaneous tumor tissues generated in (**c**). The data are presented as the means ± SD. **g** Measurement of SGOC pathway metabolites in subcutaneous tumor tissues obtained from (**c**). The data are presented as the means ± SD. *P < 0.05; **P < 0.01. **h** Relative ratios of reduced to oxidized glutathione (GSH/GSSG) in subcutaneous tumor tissues from (**c**) determined by LC–MS/MS. **i** Tumor growth curves of tumors derived from DLD-1 cells that were subcutaneously injected into nude mice (*n* = 6). Mice were treated with or without indicated amount of selumetinib. Tumor growth curves are shown. The data are presented as the means ± SD. **j** Representative IHC staining for ILF3, PHGDH, Ki-67 and cleaved-Caspase-3 in tumor tissues from (**h**). Scale bars represent 50 μm.

To determine the in vivo functional contribution of ILF3 to tumorigenesis, we performed CRC xenograft mouse model experiments. DLD1 and HCT-116 cell lines ectopically expressing ILF3 were subcutaneously implanted into nude mice. Mice bearing cells overexpressing ILF3 had accelerated tumor growth, with concurrent increased SGOC gene expression in tumor tissues (Fig. 6a, b). Moreover, knockdown of ILF3 expression with shILF3 significantly reduced tumor growth in vivo and, correspondingly, these tumors exhibited a marked reduction in SGOC gene expression, cell proliferation and increase in apoptosis (Fig. 6c–f). Notably, ILF3-KD tumors showed a pronounced decrease in the serine, glycine and SAM biosynthesis rates and subsequent GSH/GSSG ratios in the tumor mass (Fig. 6g, h).

The EGFR/ERK pathway is often deregulated in CRC; therefore, a MEK inhibitor is frequently used in treatment regimens. We observed that the MEK inhibitor selumetinib inhibited tumor growth, with concurrent reduction in the expression levels of both ILF3 and PHGDH (Fig. 6i, j). These results demonstrate that ERK inhibition was correlated with reduced ILF3 protein abundance and decreased expression of a rate-limiting enzyme in the SGOC pathway—PHGDH.

**Fig. 7:**
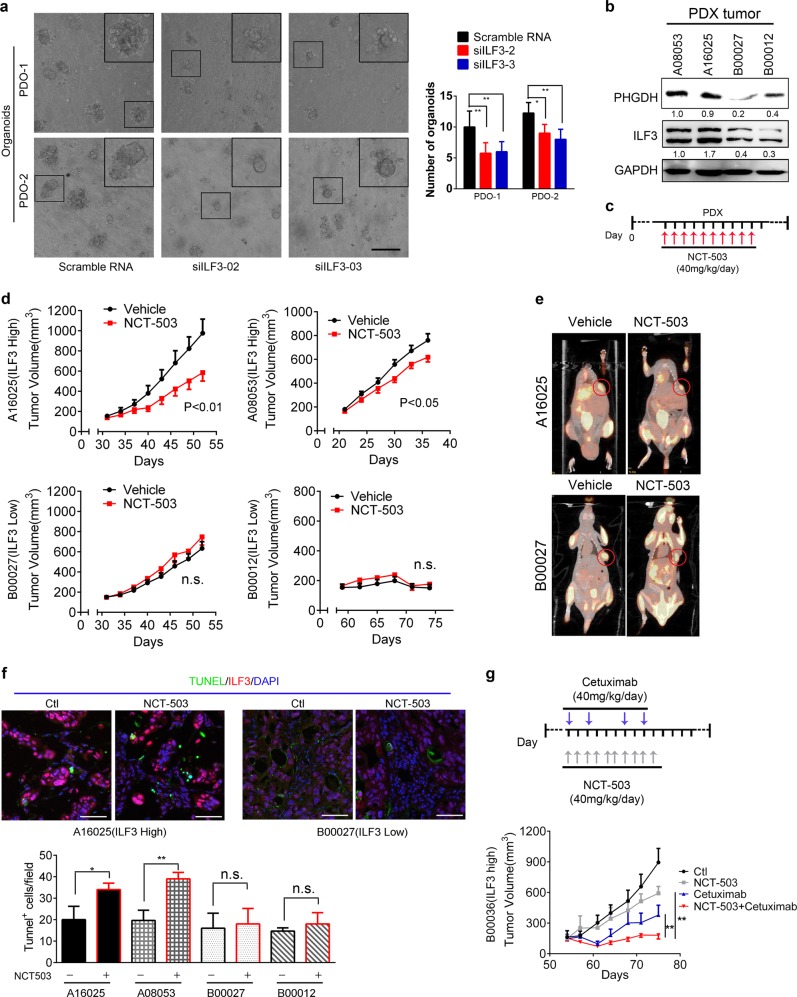
Impeding the ILF3–SGOC axis suppresses CRC malignant progression in PDO and PDXs. **a** Bright-field images and quantification of organoids after 14 days of siRNA transfection. Scale bar, 50 μm. **b**, **c** Expression level of ILF3 in indicated patient-derived xenografts (PDXs) (**b**), and treatment schedule of SGOC inhibitor NCT-503 is indicated (**c**). The mice were treated with NCT-503 (40 mg/kg/day) for 10 days. **d** Impact of NCT-503 on tumor growth in mice (*n* = 7/group) bearing indicated PDXs. The data are presented as the means ± SD. **e** Representative images of PDX growth monitored by PET-CT. The circles indicate PDXs. **f** Representative immunofluorescent images for TUNEL^+^ apoptotic signals in PDX tumors and quantitation of apoptotic TUNEL^+^ tumor cells in PDXs after NCT-503 treatment. Scale bars, 50 μm. TUNEL^+^ cells were counted and presented as a bar graph. The data are presented as the means ± SD. **g** Impact of indicated treatments on tumor growth of PDXs. The data are presented as the means ± SD. Treatment schedule of cetuximab and/or NCT-503 is indicated.

To explore whether the ILF3–SGOC axis contributes to the capacity of tumor formation, we established two more patient-relevant models, patient-derived organoids (PDO) and patient-derived xenografts (PDX).^47^ We cultured organoids in vitro and found that knockdown of ILF3 expression with siILF3 significantly reduced organoid formation (Fig. 7a). Also, we implanted fresh primary tumor samples resected from CRC patients into the immunocompromised mice and detected the ILF3 expression levels (Fig. 7b). Significantly, administration of the SGOC pathway inhibitor NCT-503 in the established ILF3-high PDX tumors (A16025, A08053) attenuated tumor progression. By contrast, SGOC inhibition had a minimal impact on the growth of ILF3-low PDX tumors (B00027, B00012) (Fig. 7c–f). The anti-EGFR monoclonal antibody cetuximab is frequently used to treat CRC patients. However, among these patients, only half respond well, suggesting a potential feedback activation of other oncogenic signals. Inhibition of BRAF (V600E) leads to a feedback to reactivate the EGFR signaling; therefore, a combination of the BRAF inhibitor encorafenib and cetuximab improves treatment efficacy in CRC.^48,49^ We deduced that targeting EGFR–ERK activation (cetuximab) and ILF3–SGOC (NCT-503) might have a synergistic effect. Next, we set up a CRC PDX model for drug efficacy testing and found that the combination of cetuximab and NCT-503 was more efficient in inhibiting tumor growth than cetuximab or the SGOC inhibitor alone (Fig. 7g; Supplementary information, Fig. S8a–c).

Together, our data demonstrates that the role of ILF3 in activating the SGOC pathway and promoting tumorigenesis can be recapitulated in vivo. We additionally elucidated the crosstalk among the EGFR–ERK signaling, ILF3 expression level, and the SGOC network during cancer formation. Our tumor model experiments also point out that inhibiting EGFR–ERK function and abrogating ILF3/SGOC activity in tumors are effective for metabolism-targeted therapies.

## Discussion

Metabolic reprogramming is identified as a hallmark of cancers because of the high energetic/anabolic needs for tumor cell growth. Deregulations of tumor suppressor genes or oncogenes elicit abnormal cellular bioenergetics that give cancer cells a growth advantage by providing fuel, building block, or changing gene expression landscape for cancer cell growth. Our clinical data, based on transcriptomic and IHC analysis, indicates that ILF3 is a prognosis-associated marker that is upregulated in colon cancer. The mechanism by which ILF3 mRNA is elevated in CRC remains to be determined. It has been reported that ILF3 mRNA level is regulated by MiR-590-5p.^48^ It is possible that deregulation of transcription factors or microRNAs may be involved. In searching for a potential impact behind ILF3 overexpression in CRC, we found that deregulation of SGOC genes to be the culprit. The signaled ILF3 overexpression-facilitated metabolic reprogramming is abnormally regulated through activated EGFR/ERK signaling, which mediates ILF3 phosphorylation and suppresses SPOP-mediated ILF3 degradation, thereby potentiating expression of SGOC genes involved in serine biosynthesis.

The roles of SGOC metabolic processes in fetal growth, bacterial infection, and immune activation have been uncovered, leading to significant medical advances. Nevertheless, the ability to selectively target SGOC metabolism for therapeutic applications remains a challenge. The obstacle has been a lack of understanding of the specific ways in which SGOC metabolism is altered in disease and how non-SGOC intermediates may contribute to pathogenesis.^8^ It is then critical to have a thorough understanding about the regulation of the SGOC pathway. A recent study revealed that Kras-driven mouse models of pancreatic and intestinal cancers were less responsive to depletion of serine and glycine, reflecting the ability of activated Kras to increase the expression of enzymes that are part of the serine biosynthesis pathway.^11^ However, how Kras activates the SGOC pathway remains unclear. Here, we show that the EGF–RAS–MAPK pathway activation stabilizes ILF3, which in turn promotes SGOC through the ability of ILF3 to bind and stabilize the mRNA of SGOC genes directly (Supplementary information, Fig. S8d), providing a mechanistic insight into this Kras-associated issue.

ILF3 can regulate the stability of RNA, but its targets are not fully characterized. Its impact on the stability of SGOC gene mRNAs is elucidated for the first time. As for regulating the stability of mRNAs, it remains to be investigated how ILF3 preferentially and specifically regulates SGOC gene expression. It could be due to the high affinity of the binding between ILF3 and SGOC gene mRNAs, as the competition may happen. For example, a previous report shows that ILF3 binds circRNA in the nucleus and viral mRNA can compete with circRNA for ILF3 binding.^35^

It is worthwhile to point out that ILF3 is involved in inhibiting MicroRNA-7 maturation, compromising MicroRNA-7’s role as an anti-oncogenic miRNA targeting EGFR.^49^ Thus, it is possible that a positive feedback loop between EGFR and ILF3 stability regulation is operated during the tumorigenesis affected by serine metabolism deregulation.

Post-transcriptional regulation of ILF3 has not been elucidated before. We show that the MATH domain of SPOP-Cul3 ubiquitin ligase interacts with evolutionarily conserved SBC motif of ILF3 for the first time. Moreover, we first identified that Erk2 mediates ILF3 S382 phosphorylation and thereby diminishes binding between ILF3 and SPOP, thereby stabilizing ILF3. The note is supported by the observation that ILF3 S382D phosphor-mimetic mutant decreases its interaction with SPOP. How this ILF3 S382 phosphorylation reduces SPOP binding warrants further investigation. It could be due to the structure conformation change after phosphorylation, as the conformation may be critical for binding. The MATH domain of SPOP-Cul3 ubiquitin ligase interacts with SBC motifs of substrates. As the SBC motifs from known SPOP substrates are virtually similar in detail, we generated a model structure of the central shallow groove of the MATH domain that binds the SBC motif. We paid attention to the missense mutations in SPOP substrate-binding (MATH) domain from CRC patients, as the significance and functional impact of these mutants have not been elucidated. We show that these cancer-derived SPOP mutants fail to earmark ILF3 for poly-ubiquitination and subsequent degradation, thereby accumulating ILF3 to subsequently activate the SGOC pathway and metabolic reprogramming. It is worthwhile to mention that prostate cancer patients have 10%–15% mutations in SPOP MATH domain.^42^ These specific SPOP MATH mutations of prostate cancer are different from those sites of CRC described in our study. However, it needs to be emphasized that these CRC mutations (R70Q, R138C, L142I) like those mutations found in prostate cancer are clustered in central shallow groove of the MATH domain that binds to SBC of ILF3 (Supplementary information, Fig. S7f), thus compromising its impacts on degrading oncogenic ILF3 protein. These data suggest that the structure determines the function. Although the mutation rate of *SPOP* gene is not high in CRC based on the TCGA data, the defect could be at the protein level. Indeed, we have found that SPOP level is low in about 50% of CRC samples as demonstrated in 270 CRC tissue microarrays (Supplementary information, Fig. S6f). Together, we think that either alteration of SPOP via mutations or low expression reduces SPOP’s tumor suppressive impacts.

Notably, we show that increased ILF3-mediated SGOC gene stability and expression confers a metabolic vulnerability to selectively target ILF3-high cancer with SGOC inhibitors. These results highlight the critical SPOP–ILF3–SGOC axis deregulation that occurs during tumor development and illustrate the potential of exploring this axis to control serine biosynthesis deregulation by reversing metabolic reprogramming. Our findings in animal experiments including PDX studies indicate that the role of ILF3 in promoting cell proliferation and serine biosynthesis can be recapitulated in vivo, thereby providing a rationale for combining EGFR/ERK signaling inhibitors (to inhibit the ERK–ILF3 axis) with SGOC pathway inhibitors (to hinder the impact of the SPOP loss-of-function due to mutations or low SPOP expression) to establish a better treatment regimen.

Further studies are needed to fully illustrate the other functions of ILF3 in CRC. Our pathway enrichment analysis revealed that ILF3 could affect several important oncogenic pathways. The impacts of those pathways, including the citrate cycle, glutamate metabolism, protein processing in endoplasmic reticulum, and the AMPK signaling, are still largely uncharacterized. This is possibly due to the various targets and functions of ILF3. Our preliminary studies showed that ILF3 protein expression levels were accumulated by methionine deprivation, suggesting that the effects and biological importance of methionine sensing with ILF3 expression warrants further investigation. Moreover, ILF3 levels were decreased by ER stress inducer tunicamycin; therefore, relationships among ILF3 and ER stress and unfolded protein response also deserve further study. More studies are needed to fully characterize the multi-layered and complex role of ILF3 in cancer cells. In summary, this study elucidates the complicated control of SGOC cancer metabolism by identifying EGF–ILF3 as a new regulatory axis of serine/glycine metabolism. Our findings suggest that ILF3 could be a therapeutic target of cancer metabolism-targeted therapies.

## Materials and methods

### Patients and tissue samples

Fresh frozen paired samples of primary CRC and adjacent normal colon tissue were collected from the Department of Surgery at the Sixth Affiliated Hospital of Sun Yat-sen University. All patients had stage II or stage III disease at the time of specimen collection. We also obtained paraffin-embedded samples of primary colorectal adenocarcinomas (prepared as TMA) from three independent CRC patient cohorts: (1) 79 patients from the Sixth Affiliated Hospital of Sun Yat-sen University (the testing cohort), (2) 270 patients from the First Affiliated Hospital of Sun Yat-sen University (the validation cohort 1) and (3) 134 patients from the 150th Central Hospital of the Chinese people’s Liberation Army (the validation cohort 2). The original immunohistochemistry slides were scanned by Aperio Versa (Leica Biosystems) which captured digital images of the immunostained slides. The Genie calculates an H-score for regions selected by the pathologist. The receiver operating characteristic curve was used to define the cut-off point. All samples were collected with the patients’ written informed consent and approval from study center’s Institutional Review Board.

### Cell culture, reagents and transfection

All the cells were obtained from ATCC, and maintained at 37 °C and 5% CO_2_. DLD-1 and HCT-8 cells were maintained in RPMI 1640 medium (RPMI) supplemented with 10% (v/v) fetal bovine serum (FBS). 293T, RKO, HT29, WiDR and SW620 cells were cultured with Dulbecco’s modified Eagle’s medium media with 10% FBS. All transient transfections of plasmids and siRNA into cell lines followed the standard protocol for Lipofectamine 2000 Transfection Reagent (Thermo Fisher, #11668019).

### shRNA knockdown of ILF3

We screened four hairpin shRNAs targeting human ILF3 transcripts and found two independent sequences that reduced mRNA levels by > 70%. These shRNAs were in the pLKO.1 vector (#31 and #32). #35 is designed to target 3’UTR of ILF3.

To produce lentiviral particles, 1 × 10^7^ HEK293T cells in a 55-cm^2^ dish were co-transfected with 10 μg pLKO.1 shRNA construct, 5 μg of psPAX2 and 5 μg pMD2.G. The supernatant containing viral particles was harvested at 48 and 72 h after transfection, and was filtered through Millex-GP Filter Unit (0.45 μm pore size, Millipore). To infect cancer cells with lentivirus, cells were infected twice with culture medium containing 2 mL lentivirus, 200 μL FBS and 5 mg/mL polybrene (Sigma) at 37 °C for 24 and 48 h. To increase the knockdown efficiency, infected cells were under several days of puromycin selection.

### Immunoprecipitation and immunoblot

For ILF3 immunoprecipitation, cells were lysed in NP-40 buffer containing 50 mM Tris–HCl (pH 7.5), 0.1% Triton-100, 1 mM EDTA, 150 mM NaCl, 0.1% Nonidet P-40, and protease inhibitor cocktail and phosphatase inhibitor (Selleck). Cell lysates (500 μL) were incubated with anti-ILF3 antibody (Abcam, ab92355) or control IgG (Sigma, I5006) overnight at 4 °C. Protein A/G agarose beads (50 μL, Santa Cruz, SC-2001) were added to each sample. After 3 h, the beads were washed five times with NP-40 buffer, followed by immunoblotting. For Flag-immunoprecipitation, cell lysates were incubated with anti-Flag M2-beads (Sigma, A2220) for 3 h at 4 °C. Immunoprecipitated proteins and phosphorylated peptides/residues were performed by Shanghai Applied Protein Technology Co., Ltd.

For the ubiquitination assay, 293T cells were cotransfected with the indicated plasmids. Nickel-nitrilotriacetic acid (Ni-NTA) agarose (Qiagen, Inc.) was used to pull down poly-ubiquitinated ILF3 proteins. After 48 h of transfection, cells were treated with 20 μM MG132 for 6 h and subjected to lysis in phosphate-buffered saline (PBS) solution containing 1% Nonidet P-40 and 10 mM imidazole. Ni-NTA-agarose beads were then incubated with cell extracts at room temperature for 4 h. Precipitates were washed three times with PBS containing 1% Nonidet P-40 and 20 mM imidazole and boiled in 1 × loading buffer. Protein complexes were then separated on SDS–polyacrylamide gels and probed with anti-Flag antibody to reveal poly-ubiquitinated ILF3. Proteins were immunoblotted following standard protocol. Antibodies specific for ILF3 (Abcam, ab92355), SPOP (Proteintech Group, 16750-1-AP), Flag-Tag (Sigma,F1804), HA-Tag (C29F4) (Cell Signaling, #3724S), Myc-Tag (9B11) (Cell Signaling, #2276S), PHGDH (Sigma, HPA021241), Ki-67 (8D5) (Cell Signaling, #9449), and GAPDH (Proteintech Group, 10494-1-AP) were purchased from the indicated companies.

### Metabolic analysis

For metabolic analysis, three technical replicates were run for each sample. More specifically, DLD1 cells (6 × 10^5^) were cultured in glucose/serine free RPMI-1640 with 10% dialyzed FBS, supplemented with 11 mM ^13^C_6_-gluose or 0.4 mM ^13^C_3_-Serine for specified intervals. Cells were treated according to our previously reported method.^50^ Briefly, cells were rinsed twice with PBS and extracted with a mixture solvent including acetonitrile, water and formic acid (80:19:1, v/v/v) on dry ice. Cells were then detached, subjected to 2 freeze–thaw cycles and centrifuged for 5 min at 13,000 rpm. Next, 5 μL, 0.03 mg/mL internal standard, 4-Cl-phenylalanine, was added to the precipitate and then re-extracted with methanol, and supernatants were pooled in a tube for evaporation under N2 evaporator.

The dried residues were performed to a derivatization reaction using 5-(diisopropylamino)amylamine (DIAAA).^51^ In brief, the prepared samples were sequentially mixed with 5 μL HOBt, 5 μL DIAAA-TEA solution, and 5 μL HATU followed by 1 min incubation at room temperature. Finally, 35 μL acetonitrile was added to make up to the final volume of 50 μL. Samples were then analyzed by ultrahigh performance liquid chromatography-quadrupole time-of-flight mass spectrometry (UHPLC-Q-TOF/MS), which was performed using an Agilent 1290 Infinity LC system and an Agilent 6550 UHD accurate-mass Q-TOF/MS system with a dual Jet stream electrospray ion source. The instrument was operated in positive ion mode and [M + H] + species were measured. Data analysis was conducted with the MassHunter Workstation Data Acquisition, Agilent MassHunter VistaFlux Software and Agilent Metabolite ID Software.

For isotopomer labeling analysis by LC-MS, the cell pellet was lysed and protein levels were measured for normalization purposes. Targeted measurement were performed using a Dionex UltiMate 3000 LC System (Thermo Scientific) coupled to a Q Exactive Orbitrap mass spectrometer (Thermo Scientific) operated in negative mode as described previously.^52^ For calculation of the total carbon contribution in ^13^C-tracing experiments, we corrected for naturally occurring isotopes (Putiande Biotechnology Corporation, Guangzhou, China).The metabolites were identified based on the standards, MS/MS spectra, and the metabolites database METLIN (https://metlin.scripps.edu/index.php).

### SAM determination

SAM determination was performed using Bridge-It^®^ S-adenosyl methionine (SAM) fluorescence assay (FM-75-506, Mediomics) according to manufacturer’s instructions.

### Xenograft colorectal cancer model

This study was approved by the Animal Ethical and Welfare Committee of Sun Yat-sen University. The in vivo tumor growth of DLD1 cells transduced with a non-targeting hairpin or shILF3-31 or shILF3-32 was determined using a subcutaneous transplant xenograft model. DLD1 (5 × 10^6^ cells/mouse) cells were inoculated subcutaneously into the hind-flanks of 5-week-old female BALB/c-nu/nu mice. After 6 days, palpable tumors had developed (~80 mm^3^), and mice were divided into 2 groups at random. After 8 days, tumors reached an average size of ~150 mm^3^. NCT-503 (40 mg/kg/day) was administered by intraperitoneal injection. Tumor length and width were measured twice weekly, and the volume was calculated according to the formula (length × width^2^)/2.

For the patient-derived xenograft (PDX), PDX models were obtained from Nanjing Biomedical Research Institute of Nanjing University and the Sixth Affiliated Hospital of Sun Yat-sen University. Patient-derived tumor fragments (3–4 mm^3^) were surgically xenografted under the skin of male NSG mice. When tumors reached approximately 100 mm^3^, mice were assigned randomly into one of two/four treatment groups.

### Organoid culture

Human cancer tissues were grown into organoids as previously described.^53^ Fresh CRC tumor tissue samples cut into small pieces, washed with ice-cold PBS, and subsequently digested with EDTA. Following Matrigel polymerization (10 min at 37 °C), Advanced DMEM/F12 is supplemented with penicillin/streptomycin, 10 mM HEPES, 2 mM GlutaMAX, 1 × B27, 1 × N2 (Life Technologies), 10 nM gastrin I (Biogems), and 1 mM N-acetylcysteine (Sigma). The following factors were used: 50 ng/mL recombinant EGF, 100 ng/mL recombinant Noggin (Peprotech), 500 nM A83-01 (Biogems), 500ng/mL R-spondin-1(Peprotech), 10 μM Y-27632 (Abmole), 10 mM nicotinamide (Sigma) and 10 μM SB202190 (Sigma).

### Immunohistochemistry

The expressions of ILF3, PHGDH, Cleaved-Caspase 3 and Ki-67 in tumors were characterized by immunohistochemistry using specific antibodies. Briefly, tumor sections (4 μm) were dewaxed in xylene, hydrated in descending concentrations of ethanol, immersed in 0.3% H_2_O_2_–methanol for 30 min, washed with phosphate-buffered saline, and probed with monoclonal anti-ILF3 (1:250), anti-PHGDH (1:200), anti-Cleaved-Caspase 3 (1:100) or Ki-67 antibodies (1:100) or isotype control at 4 °C overnight. After washing, the sections were incubated with biotinylated goat anti-rabbit or anti-mouse IgG at room temperature for 2 h. Immunostaining was visualized with streptavidin/peroxidase complex and diaminobenzidine, and sections were then counterstained with hematoxylin.

### Immunofluorescence

Paraffin-embedded samples were sectioned at 4 mm thickness. Antigen retrieval was performed by a pressure cooker for 15–20 min in 0.01 M citrate buffer (pH 6.0) to remove aldehyde links formed during initial fixation of tissues. Then, sections were blocked in PBS containing 10% donkey serum or 2% bovine serum albumin for 1 h at room temperature. Cells for immunofluorescence were fixed with 4% paraformaldehyde for 15 min at room temperature, washed with PBS and permeabilized with 0.2% Triton X-100 in PBS for 15 min. Thereafter, cells were blocked in PBS with 2% BSA for 1 h at room temperature. After blocking, samples were incubated with primary antibodies specific for Rabbit-anti-human ILF3 (1:200) overnight at 4 °C. Incubation of Alexa Fluor-conjugated secondary antibodies (Invitrogen) was carried out for 1 h at room temperature. DAPI was then used for counterstaining the nuclei. For TUNEL assay, the slides were stained using the In Situ Cell Death Detection Kit, POD at 37 °C for 30 min, followed by incubation with Alexa Fluor-conjugated secondary antibodies (Invitrogen) for 1 h at room temperature.

### RNA immunoprecipitation

RNA immunoprecipitation was performed using Magna RIP RNA-Binding Protein Immunoprecipitation Kit (17-701, Millipore) according to manufacturer’s instructions.

### mRNA stability analysis

DLD1 cells were treated with scramble or ILF3 shRNA followed by treatment of Actinomycin D (10 mg/mL) for 0, 3, 6, 9, or 12 h followed by Trizol RNA extraction. Quantitative qRT-PCR analysis was performed and relative mRNA analysis was performed using the 2^−ΔΔ^ Ct method. mRNA levels were calibrated and compared with the 0 time point.

### mRNA expression analysis and microarray

Total RNA was extracted using TRIzol (Thermo Fisher, #15596018) reagent and reverse transcribed to cDNA. Specific gene expression was quantified using SuperReal PreMix SYBR Green (biotool, #B21203) on a LightCycler480 PCR system (Roche). All genes were normalized to β-actin.

RNA samples from 3 pairs of frozen CRC patient specimens and CRC cell lines were isolated using the TRIzol reagent. Gene expression profiles were determined using Affymetrix HG-U133 Plus 2.0 GeneChips (Affymetrix) according to manufacturer’s instructions (Accession number, GSE115200). Heat maps were generated using the Java Treeview software program (http://jtreeview.sourceforge.net; used under general public license).

### In vitro kinase assay

For Erk2 kinase assay, MAP Kinase 2/Erk2 Protein (active) was purchased from Millipore (14-550). Flag-ILF3 protein was produced with TnT Quick Coupled Transcription/Translation systems (Promega, L1170). ILF3 and Erk2 protein were incubated in kinase assay buffer (25 mM Tris, pH 7.5, 5 mM b-Glycerolphosphate, 2 mM DTT, 0.1 mM Na_3_VO_4_, 10 mM MgCl_2_, 1 mM ATP) at 30 °C for 30 min. The reaction was terminated by adding SDS sample buffer and followed by boiling for 5 min in 100 °C water bath.

### In vitro ubiquitination assay

Briefly, HEK293T cells were transfected to express Flag-SPOP, HA-CUL3 and Myc-tagged RING-box protein 1 (RBX1) to purify the SPOP–CUL3–RBX1 complex by M2 immunoprecipitation. Flag-ILF3 (produced with TnT Quick Coupled Transcription/Translation systems) was incubated with the purified SPOP–CUL3–RBX1 complex, ATP (sigma-aldrich, A7699-1G), E1 (Enzo Life Sciences, BML-UW9410) and E2 (Bostonbiochem, E2-622) enzymes and his-ubiquitin (Enzo Life Sciences, BML-UW8610) at 36 °C for 60 min. Reactions were stopped by the addition of SDS sample buffer and resolved by SDS–PAGE for immunoblotting.

### Cell metabolism measurement

OCR and ECAR in real time were monitored with the Seahorse Bioscience extracellular flux analyzer (XF24, Seahorse Bioscience). Briefly, 15,000–25,000 cells were seeded in specific 24-well plates designed for XF24 in 250 mL of the appropriate growth medium and incubated overnight. Prior to measurements, cells were washed with unbuffered medium once, immersed in 500 mL of unbuffered medium, and incubated in the absence of CO_2_ for 1 h. The ECAR and OCR were then measured in a typical 8-min cycle of mix (2–4 min), dwell (2 min), and measure (2–4 min) as recommended by Seahorse Bioscience.

### Statistics

All statistical analyses were performed using SPSS 16.0. Chi-square tests and one-way ANOVA were used to assess differences in clinical variables between the CRC cohorts. Kaplan–Meier survival analyses were used to compare survival among CRC patients based on ILF3 expression; the log-rank test was used to generate p values. Cox proportional hazards regression analyses were used to assess the effect of clinical variables on patient survival. Univariate and multivariate analysis were used to assess the influence of clinical variables on survival. Significance was defined as *P* < 0.05. Differences between groups were evaluated using a two-tailed *t* test or a Mann–Whitney rank-sum test. Paired samples were compared using a paired *t* test.

## Supplementary information

Supplementary Figure 1

Supplementary Figure 2

Supplementary Figure 3

Supplementary Figure 4

Supplementary Figure 5

Supplementary Figure 6

Supplementary Figure 7

Supplementary Figure 8

Supplementary Table 1

Supplementary Table 2
