# Supplementary material for: ILF3 is a substrate of SPOP for regulating serine biosynthesis in colorectal cancer
Source: Cell Res. 2019 Nov 26;30(2):163–78. doi: 10.1038/s41422-019-0257-1 (PMC7015059; doi:10.1038/s41422-019-0257-1)
Supplement: Supplementary file 1 — Supplementary Figure 1 [file 41422_2019_257_MOESM1_ESM.pdf]

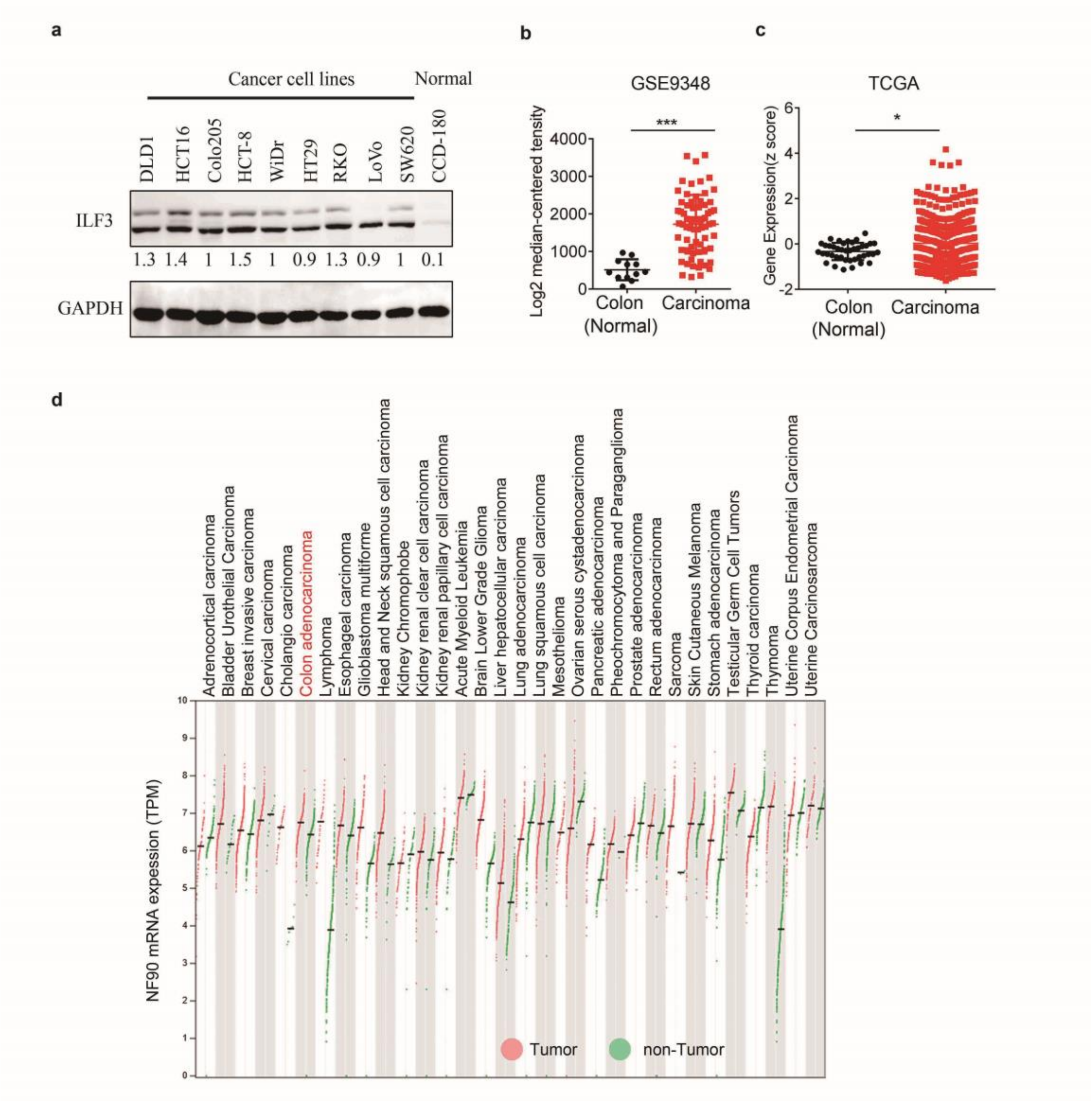

Fig. S1 ILF3 is overexpressed in cancer.

(a) Immunoblot analysis of ILF3 protein expression levels in different cell lines.

(b and c) Relative expression of ILF3 in normal and CRC tissue samples from the databases GSE9348 and TCGA.

(d) ILF3 expression (unit, transcript per million TPM) across various non-tumorous and tumorous tissues. Each dot represents information from one patient.
