# Supplementary material for: ILF3 is a substrate of SPOP for regulating serine biosynthesis in colorectal cancer
Source: Cell Res. 2019 Nov 26;30(2):163–78. doi: 10.1038/s41422-019-0257-1 (PMC7015059; doi:10.1038/s41422-019-0257-1)
Supplement: Supplementary file 2 — Supplementary Figure 2 [file 41422_2019_257_MOESM2_ESM.pdf]

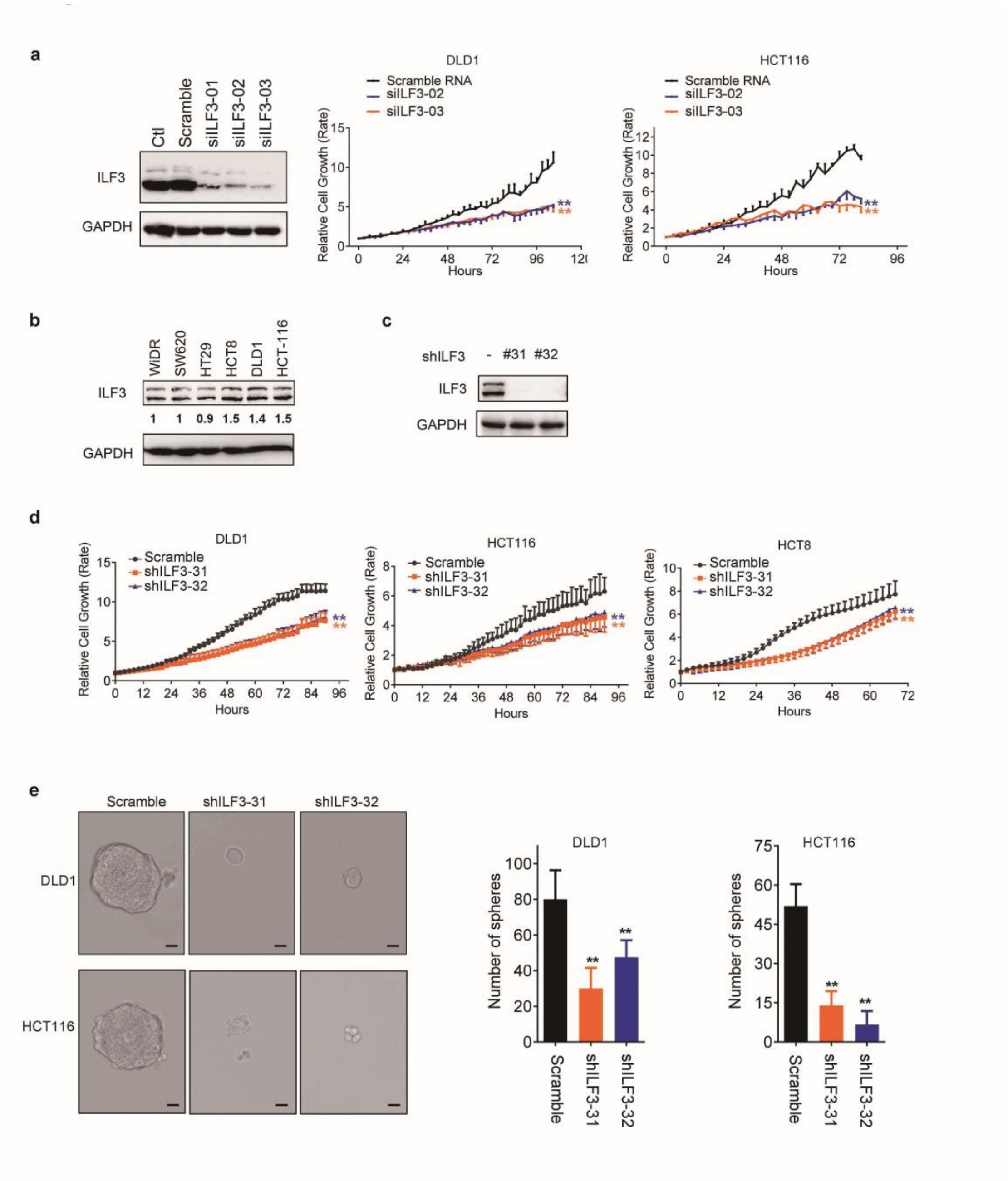

Fig. S2 Inhibiting ILF3 suppresses tumor cell growth.

(a) DLD-1 and HCT-116 cells were transfected with the indicated siRNA. Left, western blot analysis of ILF3 in cells after ILF3 knockdown with siRNA. Right, cell proliferation rates were measured. The data are presented as the means ± s.d..

(b) ILF3 protein expression in six colorectal cancer cell lines.

(c and d) Cells were infected with the indicated shRNAs. c, IB of ILF3 in cells with ILF3 knockdown via shRNA. d, Cell proliferation rates were measured. The data are presented as the means ± s.d..

(e) Representative image (left panel) and quantification of the oncosphere formation assay (right panel) at day 7. Scale bar, 25 μM. Oncosphere formation in the indicated cells infected with ILF3 shRNA.
