# Supplementary material for: ILF3 is a substrate of SPOP for regulating serine biosynthesis in colorectal cancer
Source: Cell Res. 2019 Nov 26;30(2):163–78. doi: 10.1038/s41422-019-0257-1 (PMC7015059; doi:10.1038/s41422-019-0257-1)
Supplement: Supplementary file 3 — Supplementary Figure 3 [file 41422_2019_257_MOESM3_ESM.pdf]

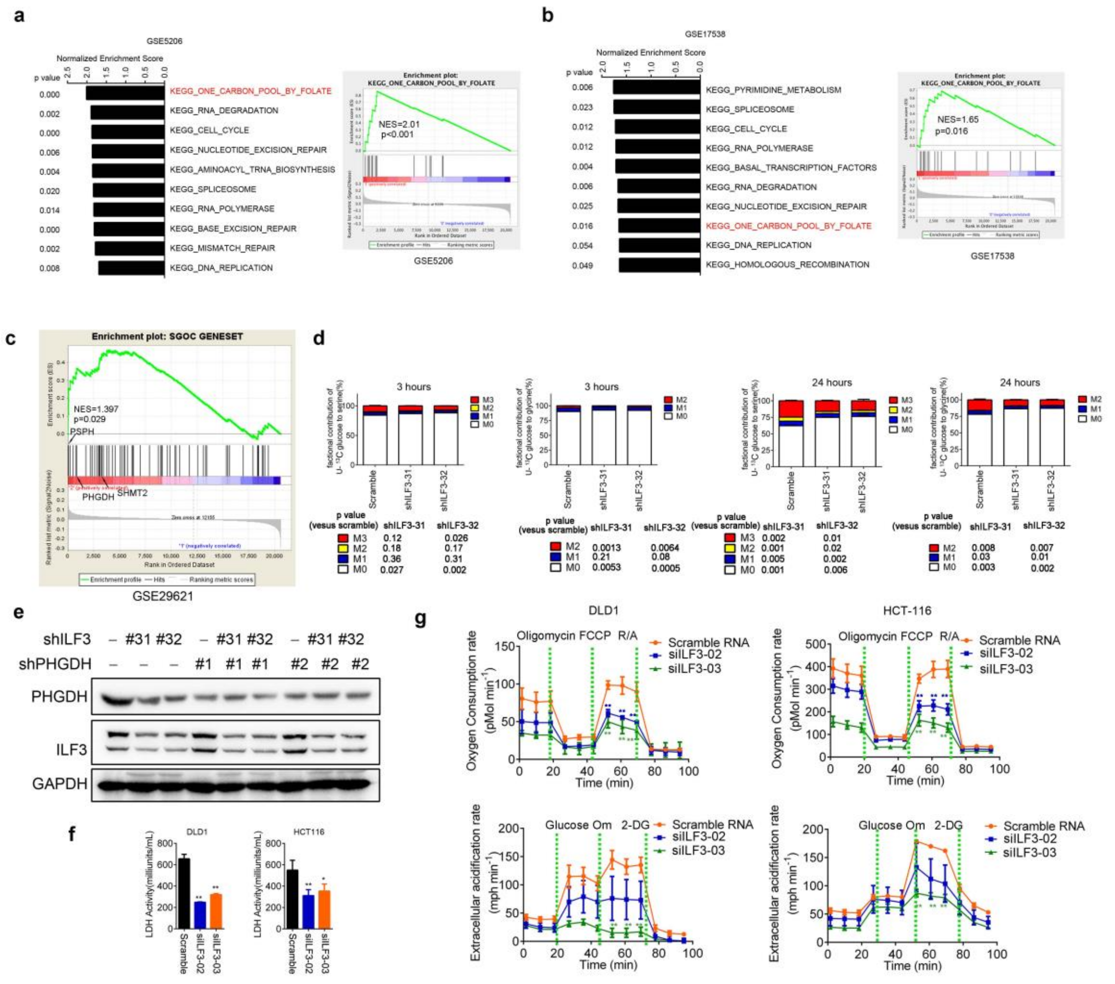

Fig. S3 ILF3 regulates cell metabolism.

(a, b) GSEA showing enrichment of KEGG-one carbon pool by folate network in the presence of high ILF3 from colon cancer set GSE5206 and GSE17538. NES, normalized enrichment score.

(c) GSEA showing enrichment of the serine-glycine-one-carbon network in the presence of high ILF3 from the colon cancer set GSE29621. NES, normalized enrichment score.

(d) Incorporation of carbon-13 (^{13}C) from [U-^{13}C] glucose (11 mM) into the indicated metabolites at 3 h and 24 h in DLD1 cells. The data are presented as the means ± s.d.

(e) Immunoblot analysis of protein expression levels in the presence of shPHGDH or shILF3.

(f) Measurement of LDH activity in DLD1 and HCT-116 cells with or without ILF3 KD.

(g) OCR and ECAR analysis of the colorectal cancer cell lines DLD1 and HCT-116 transfected with ILF3 siRNAs and scrambled siRNA.
