# Supplementary material for: ILF3 is a substrate of SPOP for regulating serine biosynthesis in colorectal cancer
Source: Cell Res. 2019 Nov 26;30(2):163–78. doi: 10.1038/s41422-019-0257-1 (PMC7015059; doi:10.1038/s41422-019-0257-1)
Supplement: Supplementary file 4 — Supplementary Figure 4 [file 41422_2019_257_MOESM4_ESM.pdf]

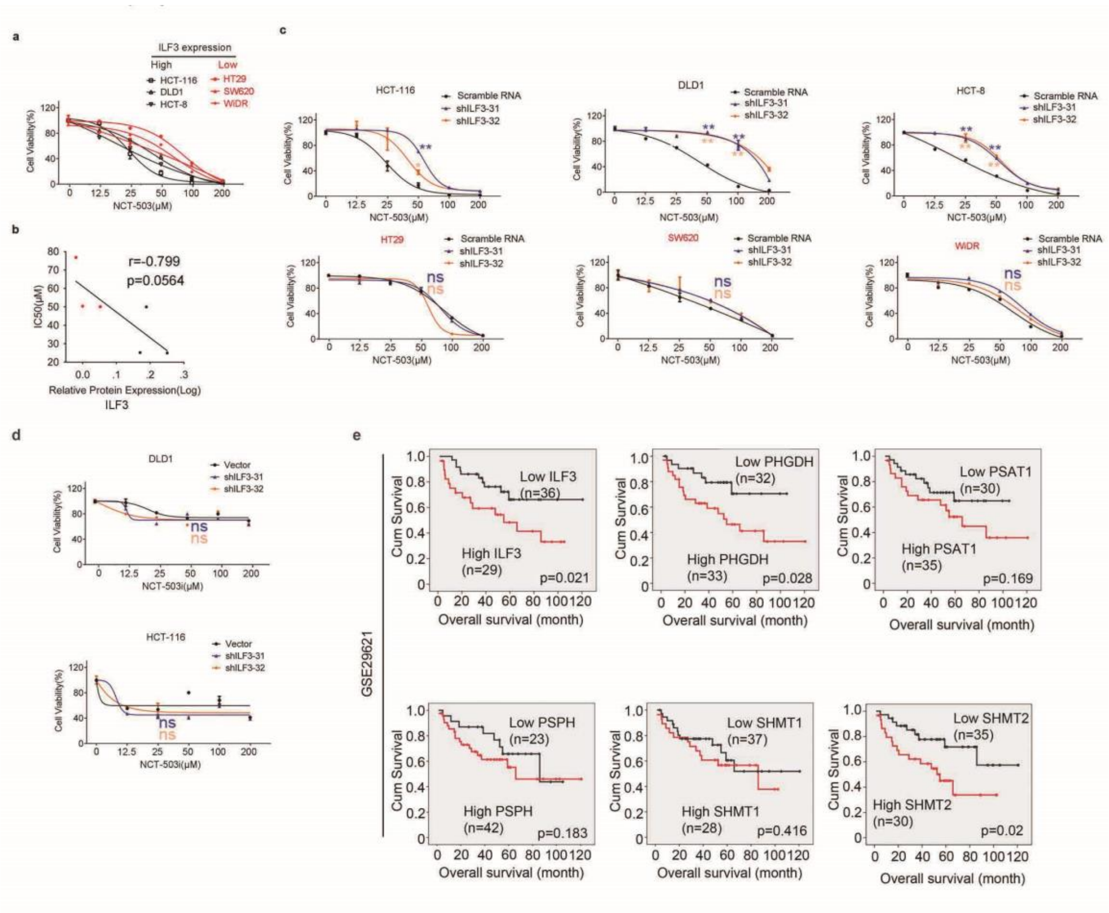

Fig. S4 ILF3 reprograms serine metabolism to maintain CRC malignant progression.

(a) Indicated cells were treated with increasing doses of NCT-503 for 48 h, and cell viability was measured.

(b) Correlation between IC50 and relative ILF3 expression. r is the Pearson correlation coefficient. Quantification was performed using ImageJ software.

(c) Cells were treated with increasing doses of NCT-503 in the presence of shILF3 for 48 h, and then, cell viability was measured.

(d) Cells were treated with increasing doses of NCT-503 inactive form (NCT-503i) in the presence of shILF3 for 48 h. Cell viability was measured.

(e) Kaplan-Meier survival curves of colorectal cancer patients from the database (GEO number, GSE29621) with low and high ILF3, PHGDH, PSAT1, PSPH, SHMT1 and SHMT2 gene expression.
