# Supplementary material for: ILF3 is a substrate of SPOP for regulating serine biosynthesis in colorectal cancer
Source: Cell Res. 2019 Nov 26;30(2):163–78. doi: 10.1038/s41422-019-0257-1 (PMC7015059; doi:10.1038/s41422-019-0257-1)
Supplement: Supplementary file 5 — Supplementary Figure 5 [file 41422_2019_257_MOESM5_ESM.pdf]

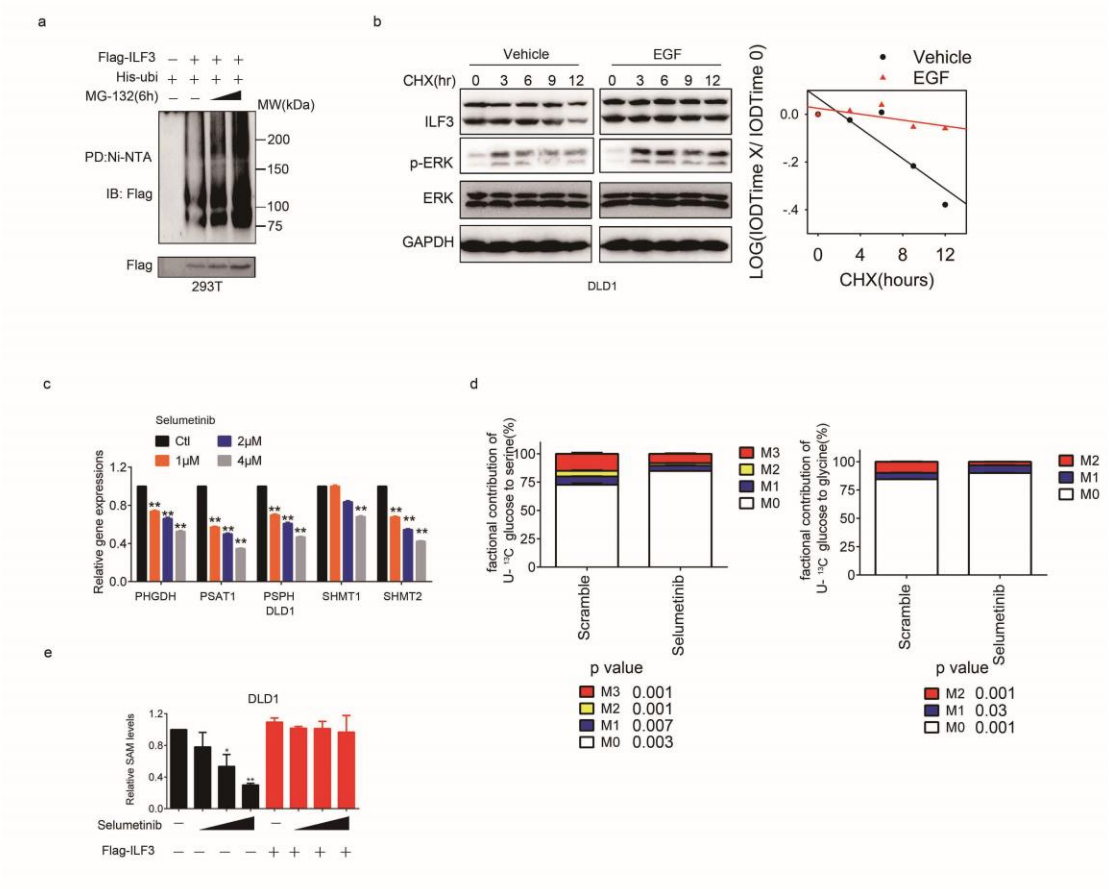

Fig. S5 The ERK pathway regulates ILF3 protein stability.

(a) Immunoblot analysis of ubiquitinated ILF3 protein in 293T cells treated with or without MG132 (20 μM).

(b) Immunoblot analysis of the ILF3 protein turnover rate in control and EGF-stimulated cells treated with cycloheximide (CHX).

(c) Serine pathway gene expression in DLD1 cells treated with selumetinib. The data are presented as the means ± s.d.

(d) Incorporation of carbon-13(^{13}C) from [U-^{13}C]-glucose into the indicated metabolites in DLD1 cells treated with selumetinib. The data are presented as the means ± SEM.

(e) SAM levels in cells treated with escalating doses of selumetinib in the presence or absence of ILF3 for 48 h.
