# Supplementary material for: ILF3 is a substrate of SPOP for regulating serine biosynthesis in colorectal cancer
Source: Cell Res. 2019 Nov 26;30(2):163–78. doi: 10.1038/s41422-019-0257-1 (PMC7015059; doi:10.1038/s41422-019-0257-1)
Supplement: Supplementary file 6 — Supplementary Figure 6 [file 41422_2019_257_MOESM6_ESM.pdf]

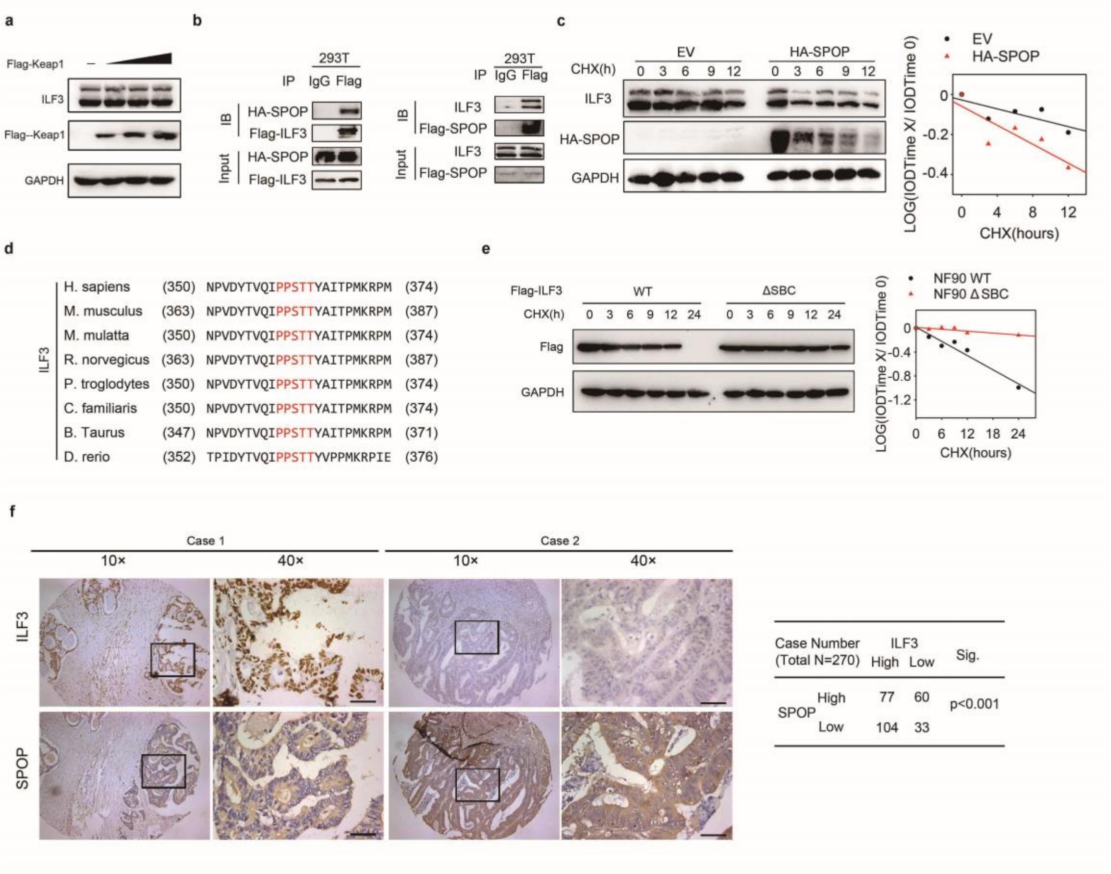

Fig.S6 SPOP is the E3 liagase of ILF3.

(a) Immunoblot analysis of ILF3 protein in 293T cells transfected with the indicated constructs.

(b) Immunoblot (IB) analysis of the indicated proteins from immunoprecipitates (IP) obtained from 293T cells transfected with Flag-SPOP (right) or HA-SPOP+Flag-ILF3 (left) in the presence of MG132 for 6 h.

(c) Immunoblot analysis of ILF3 protein in DLD1 cells transfected with the indicated plasmids, followed by the addition of 100 μg/ml cycloheximide (CHX). The density of ILF3 was measured, and the integrated optical density (IOD) was measured. The turnover of ILF3 is indicated graphically.

(d) SBC motif of ILF3 is evolutionally conserved.

(e) Immunoblot analysis of WT Flag-ILF3 protein and Flag-ILF3 (ΔSBC) in 293T cells treated with cycloheximide (CHX).

(f) Correlation of ILF3 and SPOP staining in human 270 CRC tissue microarray samples. Representative images are shown.
