# Supplementary material for: ILF3 is a substrate of SPOP for regulating serine biosynthesis in colorectal cancer
Source: Cell Res. 2019 Nov 26;30(2):163–78. doi: 10.1038/s41422-019-0257-1 (PMC7015059; doi:10.1038/s41422-019-0257-1)
Supplement: Supplementary file 7 — Supplementary Figure 7 [file 41422_2019_257_MOESM7_ESM.pdf]

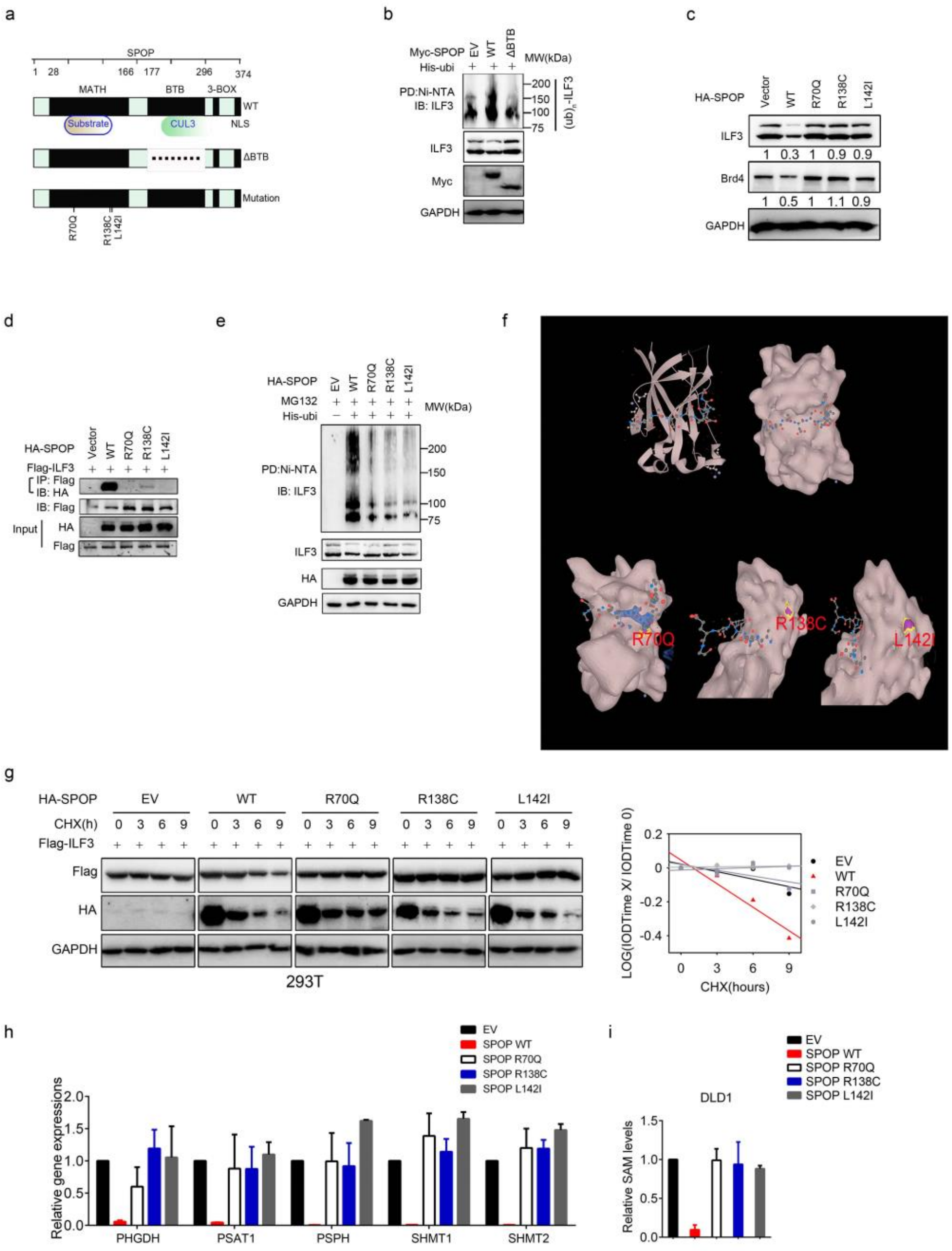

Fig. S7 Cancer type-specific SPOP mutants fail to regulate ILF3 protein stability.

(a) Schematic drawing of SPOP constructs.

(b) Immunoblot analysis of poly-ubiquitinated ILF3. Cells were transfected with the indicated constructs and then treated with MG132 for 8 h. The cell lysates were pulled down with nickel beads and immunoblotted with indicated

antibody.

(c) Immunoblot analysis of ILF3 and Brd4 expression in 293T cells transfected with the indicated constructs.

(d) Immunoblot analysis of SPOP mutant constructs from anti-Flag immunoprecipitates in 293T cells transfected with the indicated constructs and treated with 20 µM MG-132 for 6 h.

(e) Immunoblot analysis of poly-ubiquitinated ILF3 in 293T cells transfected with SPOP mutants. MG132 was added to the cells 6 h before they were harvested with guanidine-HCl-containing buffer. The cell lysates were pulled down with nickel beads and immunoblotted with an anti-ILF3 antibody. EV, empty vector.

(f) Indicated structural location of CRC-derived SPOP (including R70Q, R138C and L142I) mutants at the substrate binding groove of the SPOP MATH domain. The SBC peptide (KAASADSTTEGTPAD) is shown.

(g) Immunoblot analysis of ILF3 protein in cells transfected with the indicated constructs and treated with CHX (100 mg/ml) for the indicated time.

(h and i) Serine pathway gene expression and SAM levels after transfection with the indicated construct. EV, empty vector. The data are presented as the means ± s.d.
