# Supplementary material for: ILF3 is a substrate of SPOP for regulating serine biosynthesis in colorectal cancer
Source: Cell Res. 2019 Nov 26;30(2):163–78. doi: 10.1038/s41422-019-0257-1 (PMC7015059; doi:10.1038/s41422-019-0257-1)
Supplement: Supplementary file 8 — Supplementary Figure 8 [file 41422_2019_257_MOESM8_ESM.pdf]

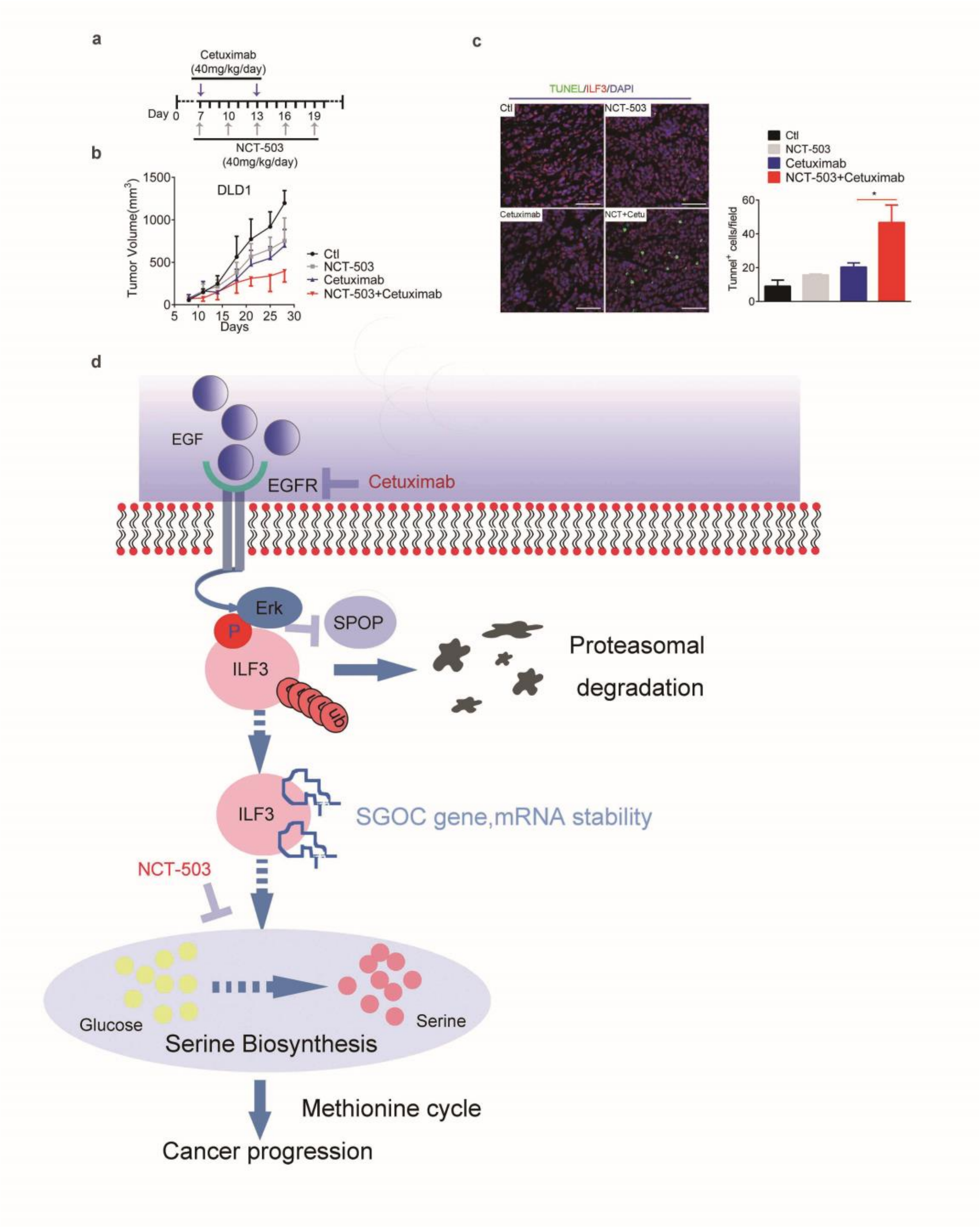

Fig. S8 Impeding the ILF3-SGOC axis suppresses CRC malignant progression.

(a) Treatment schedule of Cetuximab and/or NCT-503 is indicated. Mice (n=5/group) were treated with the indicated drugs.

(b) Impact of indicated treatments on tumor growth of DLD1 xenograft tumors.

The data are presented as the means ± s.d.

(c) Representative immunofluorescence images of $TUNEL^+$ apoptotic tumor cells (left) and quantitation of apoptotic tumor cells (right) in xenograft tumors. Scale bars, 50 μm. Signals were quantitated and presented as a bar graph. The data are presented as the means ± s.d.

(d) Model of the role of EGFR-induced ILF3 stabilization in promoting the SGOC pathway and tumorigenesis.
