# Supplementary material for: ILF3 is a substrate of SPOP for regulating serine biosynthesis in colorectal cancer
Source: Cell Res. 2019 Nov 26;30(2):163–78. doi: 10.1038/s41422-019-0257-1 (PMC7015059; doi:10.1038/s41422-019-0257-1)
Supplement: Supplementary file 9 — Supplementary Table 1 [file 41422_2019_257_MOESM9_ESM.pdf]

Table S1 Correlation between ILF3 expression and clinicopathological features of colorectal cancer patients (related to Figure 1).

	Testing cohort			Validation cohort1			Validation cohort2		
Variable	Low ILF3	High ILF3	p value[a]	Low ILF3	High ILF3	p value[a]	Low ILF3	High ILF3	p value[a]
Gender			1.000			0.001			0.848
Male	27 (64.3)	23 (62.2)		62 (66.7)	81 (45.8)		53(54.6)	21 (56.8)	
Female	15 (35.7)	14 (37.8)		31 (33.3)	96 (54.2)		44 (45.4)	16 (43.2)	
Median age			0.170			0.202			0.5626
<60 years	21 (50.0)	12 (32.4)		52 (55.9)	84 (47.5)		48 (49.5)	21 (56.8)	
≥60 years	21 (50.0)	25 (67.6)		41 (44.1)	93 (52.5)		49 (50.5)	16 (43.2)	
Histological grade			0.361			0.612			0.061
G1	8	3 (8.1)		8 (8.6)	12		8 (8.2)	4	

	(19.0)				(6.8)			(10.8)	
G2	25 (59.5)	24 (64.9)		74 (79.6)	137 (77.4)		86 (88.7)	28 (75.7)	
G3	9 (21.4)	10 (27.0)		11 (11.8)	28 (15.8)		3 (3.1)	5 (13.5)	
pT status			0.268			0.611			0.504
T1	1 (2.4)	2 (5.4)		3 (3.2)	6 (3.4)		4 (4.1)	1 (2.7)	
T2	8 (19.0)	3 (8.1)		13 (16.1)	21 (10.7)		17 (17.5)	7 (18.9)	
T3	28 (66.7)	23 (62.2)		76 (80.6)	148 (84.2)		72 (74.2)	25 (67.6)	
T4	5 (11.9)	9 (24.3)		0 (0)	3 (1.7)		4 (4.1)	4 (10.8)	
pN status			1.000			0.349			0.699
N0	19 (45.2)	17 (45.9)		57 (61.3)	116 (65.5)		60 (61.9)	22 (59.5)	
N1	23 (54.8)	20 (54.1)		36 (38.7)	61 (38.7)		37 (38.1)	15 (40.5)	
pM status			0.502			0.08			0.305

M0	38 (90.5)	31 (83.8)		89 (95.7)	151 (85.3)		95 (97.9)	35 (94.6)	
M1	4 (9.5)	6 (16.2)		4 (4.3)	26 (14.7)		2 (2.1)	2 (5.4)	
Clinical stage			0.446			0.032			0.925
I	8 (19.0)	3 (8.1)		14 (15.1)	18 (10.2)		18 (18.6)	6 (16.2)	
II	11 (26.2)	12 (32.4)		40 (43.0)	82 (46.3)		40 (41.2)	15 (40.5)	
III	19 (45.2)	16 (43.2)		35 (37.6)	51 (28.8)		36 (37.1)	14 (37.8)	
IV	4 (9.5)	6 (16.2)		4 (4.3)	26 (14.7)		3 (3.1)	2 (5.4)	

NOTE: All data are no. of patients (%).

[a]p values were calculated in SPSS16.0 using a chi-square test. p values <0.05 were considered to indicate statistical significance.
