# Supplementary material for: ILF3 is a substrate of SPOP for regulating serine biosynthesis in colorectal cancer
Source: Cell Res. 2019 Nov 26;30(2):163–78. doi: 10.1038/s41422-019-0257-1 (PMC7015059; doi:10.1038/s41422-019-0257-1)
Supplement: Supplementary file 10 — Supplementary Table 2 [file 41422_2019_257_MOESM10_ESM.pdf]

Table S2 Univariate and multivariate analyses of different prognostic parameters for colorectal cancer patients in the testing and validation cohorts (related to Figure 1).

	Univariate analysis		Multivariate analysis	
Variable	HR (95% CI)[a]	p value[b]	HR (95% CI)[a]	p value[b]
**Testing cohort (n=79)**				
Gender (male versus female)	1.467 (0.735-2.929)	0.277	0.926 (0.419 -2.047)	0.850
Age (<60 years versus ≥60 years)	1.881 (0.894-3.958)	0.096	1.934 (0.908-4.119)	0.087
Histological grade (G1 or G2 versus G3)	2.147 (1.037-4.446)	0.050	2.128 (0.938-4.828)	0.071
pT status (T1 or T2 versus T3 or T4)	2.491 (0.760-8.164)	0.132	1.459 (0.419-5.082)	0.553
pN status (N0 versus N1)	2.834 (1.315-6.108)	0.008	2.515 (1.110-5.702)	0.027

pM status (M0 versus M1)	8.490 (3.438-20.964)	<0.001	6.844 (2.533-18.489)	<0.001
ILF3 expression (low versus high)	2.638 (1.294-5.380)	0.008	2.900 (1.353-6.214)	0.006
**Validation cohort 1(n=270)**				
Gender (male versus female)	1.195 (0.752-1.900)	0.450	1.479 (0.909-2.408)	0.115
Age (<60 years versus ≥60 years)	1.595 (0.993-2.563)	0.054	2.000 (1.229-3.254)	0.005
Histological grade (G1 or G2 versus G3)	1.782 (1.009-3.150)	0.047	1.352 (0.756-2.417)	0.310
pT status (T1 or T2 versus T3 or T4)	1.908 (0.875-4.163)	0.104	1.231 (0.551-2.750)	0.613
pN status (N0 versus N1)	2.095 (1.318-3.331)	0.002	2.979 (1.834-4.839)	<0.001
pM status (M0 versus M1)	8.232 (4.961-13.660)	<0.001	9.033 (5.029-16.223)	<0.001
ILF3 expression (low versus high)	3.144 (1.606-6.155)	0.001	2.044 (1.011-4.135)	0.047
**Validation cohort 2**				

**(n=134)**				
Gender (male versus female)	0.815 (0.468-1.419)	0.469	0.818 (0.457-1.463)	0.498
Age (<60 years versus ≥60 years)	1.086 (0.630-1.870)	0.767	1.193 (0.680-2.092)	0.538
Histological grade (G1 or G2 versus G3)	1.028 (0.320-3.299)	0.964	0.957 (0.286-3.206)	0.943
pT status (T1 or T2 versus T3 or T4)	6.071 (1.889-19.507)	0.002	4.918 (1.514 -15.978)	0.008
pN status (N0 versus N1)	2.669 (1.536-4.638)	<0.001	2.129 (1.215-3.729)	0.008
pM status (M0 versus M1)	3.727 (1.325-10.486)	0.013	2.457 (0.816-7.403)	0.110
ILF3 expression (low versus high)	1.776 (1.016-3.107)	0.044	1.801 (1.015-3.196)	0.044

[a]Hazard ratios (HRs) and 95% confidence intervals (CIs) were calculated using univariate or multivariate Cox proportional hazards regression in SPSS 16.

[b]p values were calculated using univariate or multivariate Cox proportional hazards regression in SPSS 16.0. p values <0.05 were considered to indicate statistical significance.
